# Evaluation of the impact of the COVID-19 pandemic on a smoking cessation service in Derbyshire: An interrupted time series analysis

**DOI:** 10.1371/journal.pone.0303876

**Published:** 2024-06-06

**Authors:** Hayley Gleeson, Jennifer Earnshaw, Chris Craig, Chloe Hodson, Lisa Szatkowski

**Affiliations:** 1 Derbyshire County Council Public Health Department, Matlock, Derbyshire, United Kingdom; 2 Lifespan and Population Health, School of Medicine, Nottingham City Hospital, University of Nottingham, Nottingham, United Kingdom; Sheffield Hallam University, UNITED KINGDOM

## Abstract

**Background:**

Data published early in the COVID-19 pandemic suggested that smokers infected with SARS-CoV-2 were more likely to need hospital treatment or die than non-smokers, and thus this was seen as a motivator to encourage smokers to make a quit attempt. Live Life Better Derbyshire (LLBD) is an integrated lifestyle service providing free support for residents Derbyshire, UK, who want to quit smoking. On 19 March 2020, LLBD converted from offering face-to-face cessation support to a smoking cessation service delivered remotely.

**Methods:**

Interrupted time series analysis to investigate the impact of COVID-19, and the shift to remote delivery of smoking cessation support, on the number of smokers who accessed cessation support with LLBD, set a quit date, and self-reported having quit at 4-week follow-up.

**Results:**

11,393 episodes of smoking cessation support were opened with LLBD between 01 January 2018 and 31 December 2021. The weekly count of all outcomes was increasing prior to the date when LLBD converted to remote-only delivery. There was a 20% immediate drop in the number of episodes opened coinciding with this date (IRR 0.88, 95% CI 0.646–0.992) but no change in the number of quit dates set or 4-week quits or the underlying longer-term trends.

**Conclusions:**

The COVID-19 pandemic, and associated shift to remote delivery of smoking cessation support by LLBD, had no substantial sustained overall impact on measures of smoking cessation service activity and success.

## Introduction

Smokers are up to three times more likely to stop smoking when they attempt to quit using behavioural support and/or smoking cessation medications compared to unsupported quit attempts [1]. The National Institute for Health and Care Excellence (NICE) sets out clear recommendations for providing stop smoking support to individuals aged 12 and over who live in England and Wales, with services required to offer a range of accessible support options provided by trained smoking cessation advisors [2]. The availability of such support has contributed to a decline in smoking prevalence over time. In England, adult smoking prevalence has declined from 27% in 1993 to 12% in 2021 [3]. In England from April 2021 to March 2022, of the 178,198 people who set a quit date with local smoking cessation services, 55% (n = 97,654) had successfully quit 4-weeks later [4].

Data published in early 2020 at the start of the COVID-19 outbreak suggested that smokers were 2.5 times more likely to need hospital treatment or die from COVID-19 than non-smokers [5], and therefore a series of health messages were promoted nationally outlining the importance of stopping smoking. The most notable of these was the ‘Quit for COVID’ campaign [6], begun by a General Practitioner (GP) in South West England in March 2020, and picked up nationally by organisations such as Action on Smoking and Health (ASH) and the National Centre for Smoking Cessation and Training (NCSCT). This campaign, and others that followed [7], raised awareness of the consequences of COVID-19 as a smoker and, capitalising on COVID-19 as a strong personal motivator for lifestyle change, encouraged smokers to make a quit attempt.

However, the COVID-19 outbreak resulted in a national lockdown in the UK and restrictions on daily life which necessitated substantial changes in the way public services were delivered, including services to help smokers stop smoking. With individuals being instructed to stay at home, and public buildings, from where smoking cessation services were being delivered, being closed, many services shifted to remote delivery. There is little evidence whether the outbreak of COVID-19 affected demand for, and the success of, stop smoking services.

Live Life Better Derbyshire (LLBD) is an integrated lifestyle service providing free support for residents of Derbyshire, a county in the English Midlands [8]. In 2021, Derbyshire’s population was 796,847 (51.0% female), with a median age of 45.8 years [9]. In 2011 (the most recent data available), 73% of the population lived in urban areas [10]. An estimated 14.1% of the adult population in Derbyshire currently smoke [11].

Since 2018, LLBD has been run by Derbyshire County Council; prior to this it was a commissioned service provided by the NHS. LLBD provides holistic wellbeing assessments and support to stop smoking, lose weight and increase physical activity levels, as well as other signposting services. LLBD offers a free, 12 week, smoking cessation service to smokers aged 12 and over, following the National Centre for Smoking Cessation and Training (NCSCT) standard treatment programme [12]. The service offer includes remote and in-person support, access to free Nicotine Replacement Therapy (NRT) on prescription (and previously also varenicline and Bupropion prior to their withdrawal from sale in July 2021 and November 2022 respectively), and other useful tools to help smokers quit. Prior to the outbreak of COVID-19, 80% of smoking cessation service activity was delivered face to face; the remaining 20% was delivered remotely to support smokers with mobility and/or access issues. In March 2020, LLBD converted to a smoking cessation service delivered entirely remotely. A ‘Quit for COVID’ campaign was also launched in Derbyshire in June 2020 to encourage people to stop smoking.

This study uses a single-group interrupted time series analysis to investigate the impact of the change in cessation service delivery modality that occurred as a result of the COVID-19 pandemic on the number of smokers who accessed cessation support with LLBD, set a quit date, and were self-reported successful quitters at 4-week follow-up. Understanding the impact of COVID-19 on LLBD will help inform service planning in the case of future pandemics, as well as more immediately inform how best to deliver smoking cessation services for optimum impact.

## Methods

### Study design and data source

This retrospective cross-sectional study uses data captured from the electronic records maintained by the LLBD smoking cessation service. Data were included in the evaluation from all Derbyshire residents aged 12+ who opened an episode with the service from 01 January 2018 to 31 December 2021. Data were extracted on 1^st^ July 2022. Approval for this study was granted by the Derbyshire County Council research governance panel, who deemed this to be a service evaluation of routinely collected service data not requiring further ethical approval. The authors did not access any personal identifying information that could identify participants. Consent was not obtained from individual participants as data were analysed anonymously.

### Outcomes

We pre-specified three primary outcomes, based on data items nationally reported and used to measure the success of stop smoking services: a) the number of episodes opened per week; b) the number of quit dates set; and c) the number of successful quitters–service users who at the 4-week follow-up self-reported as having not smoked at all in the last two weeks [13]. We also defined two secondary outcomes: a) the number of quit dates set as a percentage of the number of episodes opened; and b) the number of successful 4-week quits as a percentage of the number of quit dates set. Data were aggregated by 7-day period, beginning on the 1^st^ January of each year (data for week 53 of each year were combined with data for week 52), and attributed to the week each initial episode was opened.

### Exposure

On 19 March 2020 the UK Prime Minister issued a statement advising people to stay at home if they had symptoms of COVID-19; full lockdown followed on 23 March 2020. LLBD changed to remote-only service delivery on 19 March 2020. Therefore, we pre-specified the weeks prior to 19 March 2020 as the pre-interruption period, 19 March 2020 as the interruption date and the weeks after 19 March 2020 as the post-interruption period.

### Variables

Data were extracted from LLBD smoking cessation service records to describe the socio-demographic characteristics of smokers accessing the service, including age group, gender, ethnicity, and Index of Multiple Deprivation (IMD) decile.

### Statistical methods

All analyses were conducted in R Studio version 4.0.3 [14]. Following methods used elsewhere [15], for each outcome segmented regression [16] was conducted using a generalised additive mixed model (GAMM) to estimate an incidence rate ratio (IRR) for the weekly change in each outcome prior to the outbreak of COVID-19, any immediate absolute change in the magnitude of each outcome between the week beginning 12 March 2020 (the last week before the pre-specified interruption point) and the week beginning 19 March 2020, and any change in trend in the post-interruption period compared to the pre-interruption period. The outcome count variables were overdispersed and so a quasi-Poisson distribution was specified. A thin plate spline was included in each model to account for seasonal variation by week of the year. Plots of the autocorrelation and partial autocorrelation functions were used to identify plausible values for autoregressive and moving average terms to include in the GAMM models to account for autocorrelation. The Akaike Information Criterion (AIC) was used to compare models with linear, quadratic, and cubic trends for the weeks after the outbreak of COVID-19, with smaller values of the AIC indicating better model fit.

### Sensitivity analysis

Service staff reported changes in service users’ behaviour in the days and weeks prior to 19 March 2020, with many not attending support sessions through fear of catching coronavirus. Many community venues also closed prior to 19 March 2020 to prevent virus transmission. To explore the potential impact of these changes we conducted an exploratory sensitivity analysis with the interruption specified 4-weeks earlier than our primary analysis.

### Subgroup analyses

We conducted pre-specified exploratory subgroup analyses to determine if there were differences in outcomes in different population groups according to: gender (male vs. female); age group (< cohort median age vs. ≥ cohort median age); IMD (five least deprived deciles vs. five most deprived deciles). We had planned a subgroup analysis by ethnic group, but on examination of the data >96% of service users were of white ethnicity, limiting the power to explore differences between white and non-white groups.

## Results

11,393 episodes were opened with the LLBD smoking cessation service between 01 January 2018 and 31 December 2021. In 64.8% of episodes opened (n = 7,381/11,393), a quit date was set. In 66.0% of episodes where a quit date was set (n = 4,871/7,381), a self-reported successful quit at 4-weeks was recorded. Table 1 describes the characteristics of the study population, the number of episodes opened, quit dates set, and successful 4-week quits, and compares these between episodes opened before and after the pre-specified interruption date of 19 March 2020.

**Table 1:** Characteristics of study population, comparing episodes opened before and after outbreak of COVID-19.

	Full cohort	Pre COVID-19	Post COVID-19	p-value
**Number of weeks**	208	115	93	
**Episodes opened**	11,393	6,218	5,175	
**Quit dates set, n (% of episodes opened)**	7,381 (64.8)	3,369 (45.6)	4,012 (54.4)	<0.001
**4-week quits, n (% of quit dates set)**	4,871 (66.0)	2,181 (44.8)	2,690 (55.2)	0.039
**Gender, n (% of all episodes opened)**				
Female	6,800 (59.7)	3,670 (59.0)	3,130 (60.5)	0.159
Male	4,563 (40.1)	2,524 (40.6)	2,039 (39.4)	
**Age group, n (% of all episodes opened)**				
< median age	5,696 (50.0)	3,106 (50.0)	2,590 (50.0)	0.918
≥ median age	5,697 (50.0)	3,112 (50.0)	2,585 (50.0)	
**Index of Multiple Deprivation, n (% of all episodes opened)**				
Most deprived	7,522 (66.0)	4,093 (65.8)	3,429 (66.3)	0.831
Least deprived	3,842 (33.7)	2,100 (33.8)	1,742 (33.7)	
**Ethnicity, n (% of all episodes opened)**				
White	10,988 (96.4)	5,990 (96.3)	4,998 (96.6)	0.464
Non-White	208 (1.8)	112 (1.8)	96 (1.9)	

Missing data: Gender, 30 (0.3%); Index of Multiple Deprivation, 29 (0.3%); Ethnicity, 197 (1.7%).

There were no statistically significant differences in the characteristics of service users between episodes opened before and after the interruption date.

**Fig 1:**
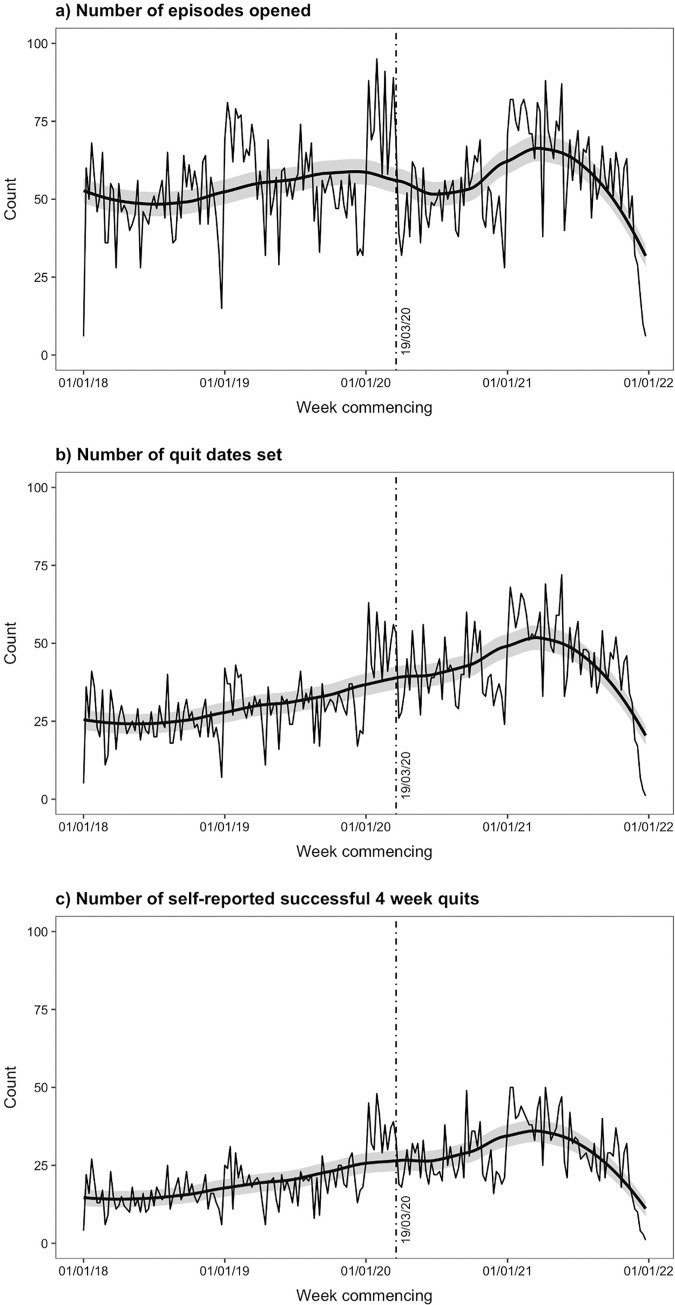
Weekly count data for the primary outcomes, overlaid with smoothed fitted regression lines.

Table 2 shows the parameter estimates for the best fitting segmented regression models for the primary outcomes and Fig 1 shows the weekly count data overlaid with fitted regression lines smoothed using a loess smoothing function with 50% smoothing span.

**Table 2:** Parameter estimates for the best-fitting segmented regression models for the primary outcomes.

	Episodes opened			Number of quit dates set			Number of 4-week quits		
	IRR (95%CI)	SE	p-value	IRR (95%CI)	SE	p-value	IRR (95%CI)	SE	p-value
Trend prior to interruption point*	1.003 (1.002–1.004)	0.001	<0.001	1.005 (1.004–1.007)	0.001	<0.001	1.007 (1.006–1.009)	0.001	<0.001
Immediate absolute change at interruption point	0.800 (0.646–0.992)	0.111	0.046	1.111 (0.874–1.411)	0.123	0.396	1.042 (0.804–1.350)	0.134	0.761
Trend post-interruption point	0.988 (0.970–1.006)	0.009	0.202	0.981 (0.961–1.001)	0.011	0.072	0.977 (0.955–0.998)	0.011	0.040
Trend post-interruption point ^2	1.001 (1.000–1.001)	0.000	0.021	1.001 (1.000–1.001)	0.000	0.013	1.001 (1.000–1.001)	0.000	0.006
Trend post-interruption point ^3	1.000 (1.000–1.000)	0.000	0.002	1.000 (1.000–1.000)	0.000	0.001	1.000 (1.000–1.000)	0.000	<0.001

Abbreviations: IRR, incidence rate ratio; SE, standard error

*Interruption point pre-specified as 19 March 2020

For all outcomes there was a week-on-week increasing trend prior to the interruption. There was evidence for a decrease in the number of episodes opened at the pre-specified interruption point (IRR = 0.800; 95% CI 0.646–0.992), but no evidence of a step change in the number of quit dates set or the number of 4-week quits. For the number of episodes opened and the number of quit dates set, there was no change in the pre-interruption upward linear trend after the interruption point. For the number of 4-week quits the pre-interruption upward trend was reduced after the interruption point. Models for all outcomes included quadratic and cubic trends in the post-interruption data, with weekly counts plateauing in Spring 2021 before declining towards the end of the study period.

**Fig 2:**
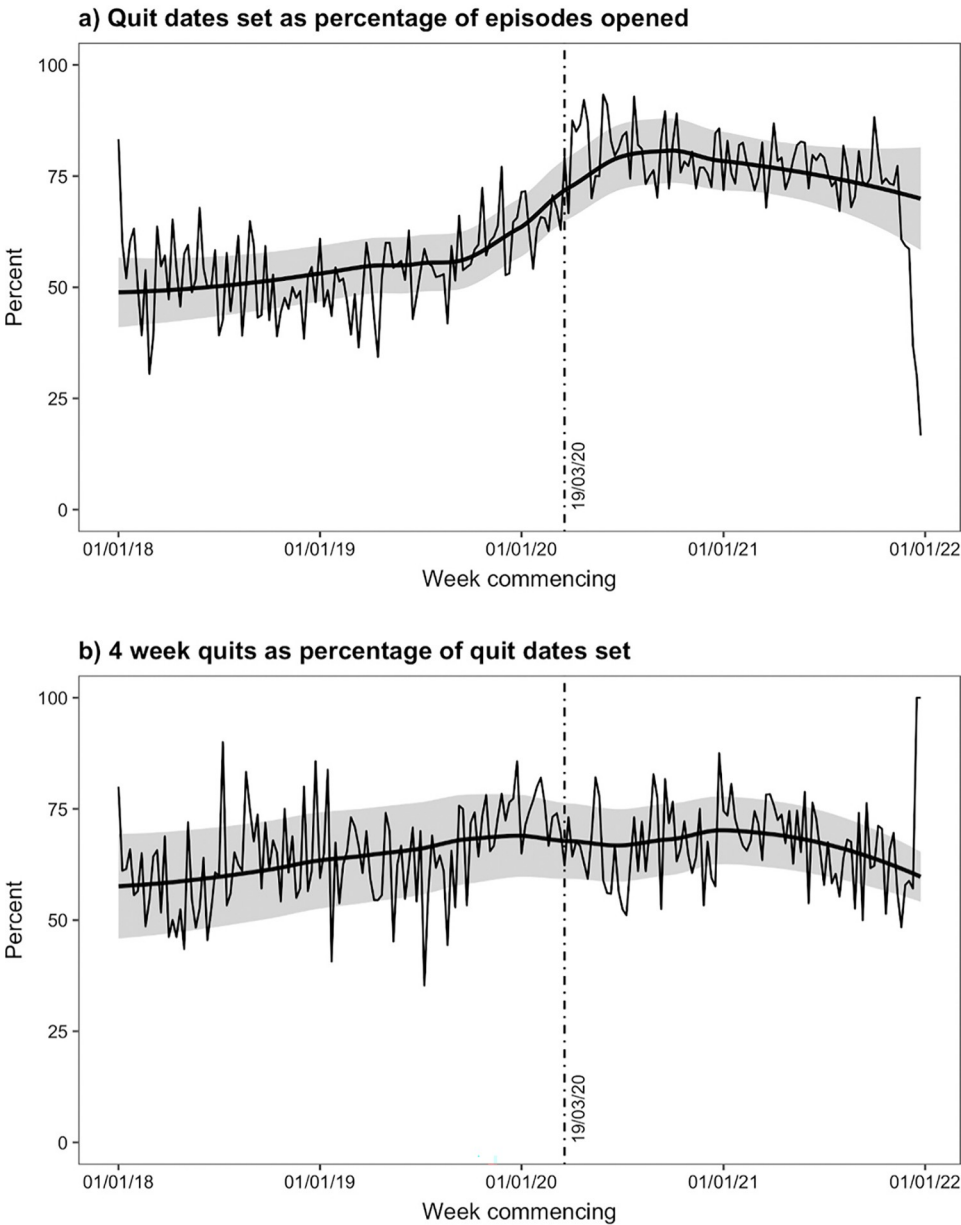
Weekly count data for the secondary outcomes, overlaid with smoothed fitted regression lines.

Table 3 and Fig 2 show the results of the secondary analysis modelling quit dates set as a percentage of episodes opened, and 4-week quits as a percentage of quit dates set. The number of quit dates set as a percentage of episodes opened was increasing prior to COVID-19, but there was an additional step change increase at the interruption point (IRR = 1.375; 95% CI 1.267–1.492) and then a slight downward trend post-interruption. There was evidence of a decrease in the number of 4-week quits as a percentage of quit dates set at the interruption point (IRR = 0.875; 95% CI 0.799–0.959).

**Table 3:** Parameter estimates for the best-fitting segmented regression models for the secondary outcomes.

	Quit dates set as a percentage of episodes opened			4-week quits as a percentage of quit dates set		
	IRR (95%CI)	SE	p-value	IRR (95%CI)	SE	p-value
Trend prior to interruption point*	1.002 (1.001–1.003)	0.000	<0.001	1.002 (1.001–1.003)	0.000	<0.001
Immediate absolute change at interruption point	1.375 (1.267–1.492)	0.042	<0.001	0.875 (0.799–0.959)	0.047	0.005
Trend post-interruption point	0.986 (0.995–0.998)	0.001	<0.001	1.003 (0.999–1.007)	0.002	0.113
Trend post-interruption point ^2	N/A	N/A	N/A	1.000 (1.000–1.000)	0.000	0.004
Trend post-interruption point ^3	N/A	N/A	N/A	N/A	N/A	N/A

Abbreviations: IRR, incidence rate ratio; SE, standard error; N/A, parameter not included

*Interruption point pre-specified as 19 March 2020

### Sensitivity analysis

S1 Table in S1 File shows the results of the sensitivity analysis specifying the interruption date 4-weeks earlier than in the primary analysis. Here there was no statistically significant evidence of a step change in the number of episodes opened at the point of interruption, but evidence of an increase in the number of quit dates set (IRR 1.357; 95% CI 1.091–1.688) and the number of 4-week quits (IRR = 1.321; 95% CI 1.042–1.674). The direction and magnitude of the parameter estimates for the secondary outcomes remained similar.

### Subgroup analysis

S2 Table in S1 File shows the results of the subgroup analyses exploring variations by gender, age group and deprivation. Statistical power was limited given the smaller counts of outcomes by week, and so results are exploratory only. There was some evidence of a larger step change decrease in the number of episodes opened amongst females than males at the point of interruption. For both the number of quit dates set and the number of 4-week quits, there was evidence of an immediate decrease in both older service users and the least deprived, but not in their respective comparison groups. There were no variations between subgroups in the number of quit dates set as a percentage of episodes opened, but evidence of an immediate decrease in both females and the least deprived in the number of 4-week quits as a percentage of quit dates set.

## Discussion

This evaluation of the impact of the COVID-19 pandemic on Live Life Better Derbyshire has found some evidence of an immediate negative impact on the number of episodes opened with the smoking cessation service, but no impact on the number of quit dates set nor the number of self-reported 4-week quits. These relative impacts may account for the apparent immediate increase in the percentage of episodes opened which resulted in a quit date being set, and a small but statistically significant reduction in the percentage of quit dates set which resulted in a successful quit.

Despite the national promotion of health messages outlining the importance of stopping smoking, there does not appear to have been any increase in the number of smokers seeking support to quit at the start of the COVID-19 pandemic; the opposite appears true in these data. However, based on the numbers of quit dates set and successful quit attempts, LLBD appears to have successfully adapted and been resilient to the widespread changes in service delivery necessitated by the COVID-19 pandemic. Our findings also suggest that the population of Derbyshire was still motivated to quit smoking at this time, despite the many impacts of the COVID-19 pandemic on health, society and the economy.

Our findings align with those of a study of the impact of the COVID-19 pandemic on the number of downloads of a popular smoking cessation app, which found no evidence of a large step change or increasing trend in downloads [15]. The decrease in the number of episodes opened at the point of interruption may be due to a combination of factors, both known and unknown. The community venues used by LLBD to deliver the smoking cessation service were closing in the build-up to the first national lockdown, which is likely to have prevented those who wanted to set a quit date from accessing the service. Service staff also reported a decrease in referrals from healthcare and social care professionals due to these professionals entering ‘crisis mode’. Given the pervasive and unprecedented nature of the COVID-19 pandemic, there may be several other personal, organisational, environmental, and societal factors that we have not been able to account for which may explain the results.

Our findings suggest that, once smokers had engaged with LLBD, there was no overall change in the effectiveness of the service as measured by the number of quit dates set and the number of 4-week quits. The decline in the number of episodes opened may represent a decline in smokers less committed to trying to quit coming forward for support to do so. These findings may also suggest that the shift to full remote delivery of cessation support had no impact on service outcomes. However, the findings of the exploratory subgroup analysis point to some potential differences in impact which should be explored further when considering how best to organise and deliver smoking cessation services post-pandemic. Other studies have likewise raised the possibility that cessation support delivered remotely may be less effective than that delivered in-person [17] but virtual support may also be beneficial for hard-to-reach groups [18]. Although <5% of quit attempts in England are made with the support of a smoking cessation service such as LLBD [19], quitting using a stop smoking service increases the chance of successful cessation threefold [20]. It is therefore vital to maximise the reach of smoking cessation services, considering how mode of service delivery can best help achieve this.

The interrupted time series methodology requires pre-specification of the interruption point, which we defined as the date on which LLBD changed to remote-only service delivery. However, smokers’ behaviours may have changed prior to this, with the emerging outbreak of SARS-CoV-2 gaining attention from December 2019 and throughout the first quarter of 2020. Our sensitivity analysis, in which we moved the interruption point 4-weeks earlier, shows significant step change increases in the number of quit dates set and the number of 4-week quits at this earlier interruption point. This suggests that there may have been some increased cessation activity prior to the date at which LLBD changed to remote-only service delivery.

Our data show declines in cessation activity from early summer 2021 to the end of the study period. Although Derbyshire residents can self-refer to the LLBD smoking cessation service, the majority of referrals for smoking cessation support are made via general practitioners (GPs). During the latter part of the study period there was an increase in the number of referrals made to the weight management aspect of LLBD, particularly from GPs, which may have displaced and reduced the number of referrals from GPs for smoking cessation support. In addition, more LLBD resources were assigned to dealing with the increase in weight management referrals in the latter half of 2021 (whereas previously smoking cessation referrals were given priority) and with the onset of the Omicron variant of SARS-CoV-2 in November 2021 there was increased staff absence due to illness. This reduced capacity may have had an impact on smoking cessation service delivery, resulting in the reductions in cessation activity observed.

The inclusion of a smoothed spline for week of the year in our regression models helps account for seasonal variation in smoking cessation activity, including the well-known New Year uptick in smokers accessing smoking cessation services. However, other factors, such as staff recruitment in early 2021 to increase service capacity, may also explain some of the trends in cessation activity seen. The withdrawal of varenicline in July 2021 may also explain some of the decline in cessation activity in the last 6 months of the study period.

In line with smoking cessation service data reported nationally, the data analysed here are based on episodes opened rather than individual smokers, and so repeat service users were included in the analysis. Of the 11,393 episodes opened in the evaluation period, 4,645 episodes were opened by service users who accessed the service more than once. Furthermore, where support was delivered remotely, data on 4-week quit status are self-reported–the service user was counted if at the 4-week follow-up they reported that they had not smoked at all in the last two weeks. Where cessation support was delivered in-person, an exhaled carbon monoxide test was used to verify the self-reported quit status. This may have resulted in an overcount of the number of successful quit attempts during the period when cessation support was delivered entirely remotely, as self-reported data tend to underestimate continued smoking compared to biological verification. This makes it difficult to isolate the impact of the shift in mode of service delivery at the start of the COVID-19 pandemic from the impact of the way in which quit status was ascertained.

This evaluation contributes to the emerging evidence base of the impact of the COVID-19 pandemic on demand for, access to, and outcomes of, local smoking cessation services. It highlights considerations for service delivery, relevant within and beyond Derbyshire, both in the case of future pandemics as well as more immediately. The remote support delivery model, used by a small proportion of service users pre-COVID, expanded to all service users during the pandemic, and which post-COVID includes an offer of both remote and face-to-face support to all service users, has enabled LLBD to maximise the accessibility of smoking cessation support delivered in Derbyshire. Further research is warranted to determine the generalisability of these findings to other smoking cessation services who were also forced to change their mode of delivery with the arrival of COVID-19, as well as the longer-term impact of these changes, both overall and within different socio-demographic population groups.

## Supporting information

S1 File Sensitivity and subgroup analyses. (DOCX)
