# Supplementary material for: Evaluation of the impact of the COVID-19 pandemic on a smoking cessation service in Derbyshire: An interrupted time series analysis
Source: PLoS One. 2024 Jun 6;19(6):e0303876. doi: 10.1371/journal.pone.0303876 (PMC11156325; doi:10.1371/journal.pone.0303876)
Supplement: S1 File — (DOCX) [file pone.0303876.s001.docx]

**Supporting information file S1: Sensitivity and subgroup analyses**

**Supplementary Table 1: Sensitivity analysis moving interruption date 4 weeks earlier than the primary analysis**

| **Number of episodes opened** |  |
| --- | --- |
| 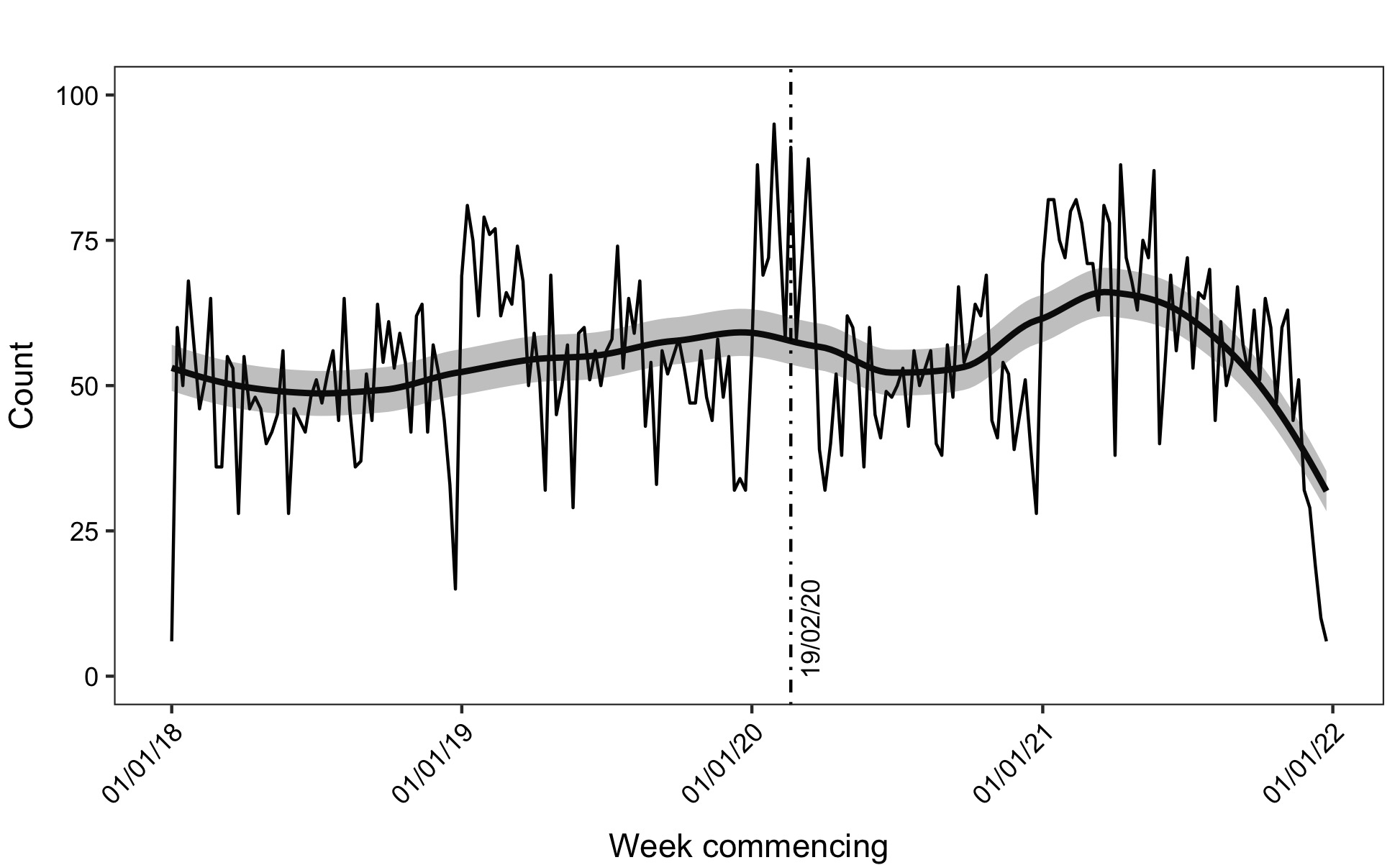   \|  \| IRR \| 95% CI \| SE \| p-value \| \| --- \| --- \| --- \| --- \| --- \| \| Time \| 1.003 \| 1.002-1.004 \| 0.001 \| <0.001 \| \| Level \| 1.076 \| 0.883-1.310 \| 0.102 \| 0.474 \| \| Slope \| 0.968 \| 0.952-0.984 \| 0.008 \| <0.001 \| \| Slope^2^ \| 1.001 \| 1.001-1.001 \| 0.000 \| <0.001 \| \| Slope^3^ \| 1.000 \| 1.000-1.000 \| 0.000 \| <0.001 \| |  |
| **Number of quit dates set** | **Quit dates set as a % of episodes opened** |
| 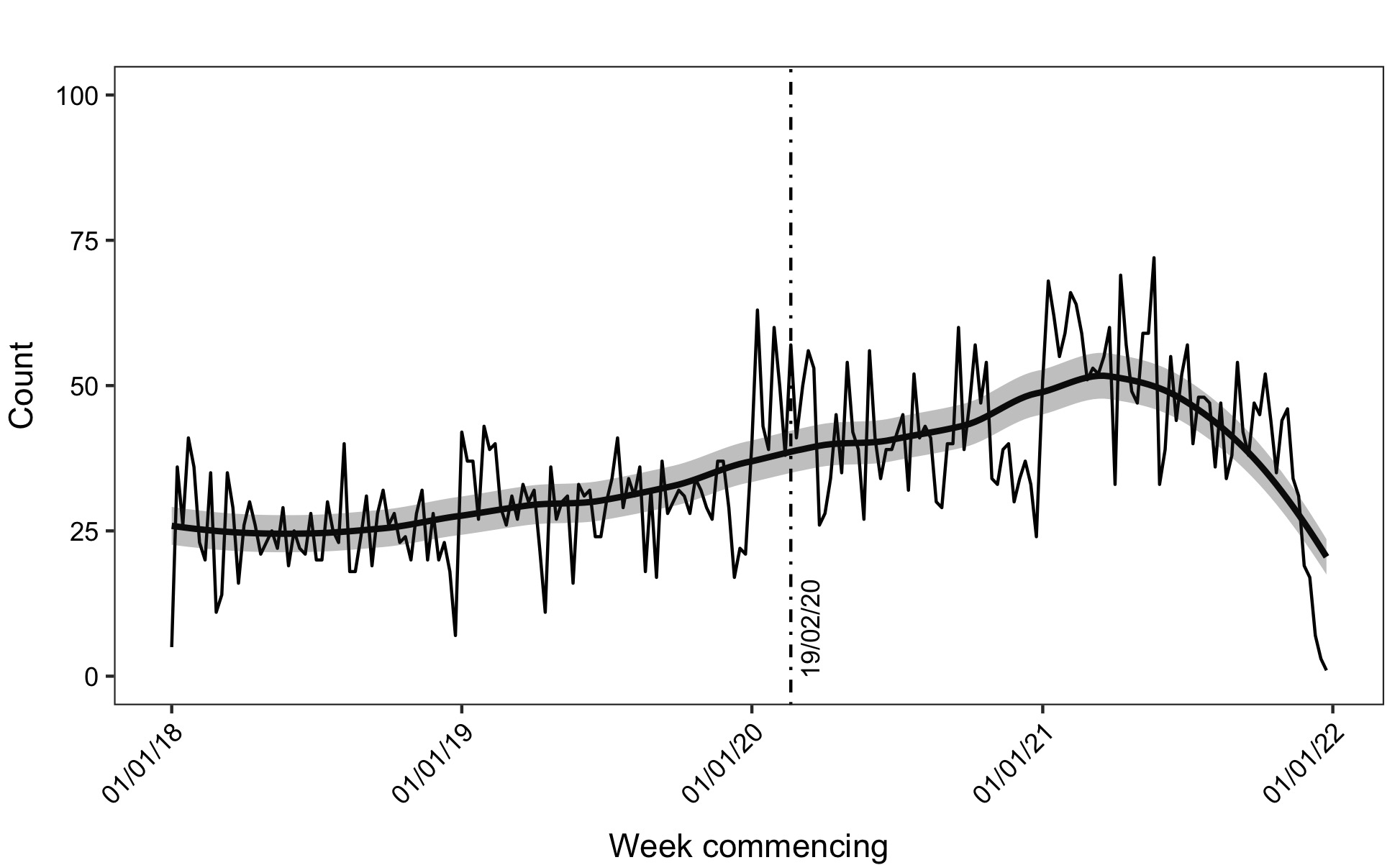   \|  \| IRR \| 95% CI \| SE \| p-value \| \| --- \| --- \| --- \| --- \| --- \| \| Time \| 1.005 \| 1.003-1.006 \| 0.001 \| <0.001 \| \| Level \| 1.357 \| 1.091-1.688 \| 0.113 \| 0.007 \| \| Slope \| 0.971 \| 0.954-0.988 \| 0.009 \| 0.001 \| \| Slope^2^ \| 1.001 \| 1.000-1.001 \| 0.000 \| <0.001 \| \| Slope^3^ \| 1.000 \| 1.000-1.000 \| 0.000 \| <0.001 \| | 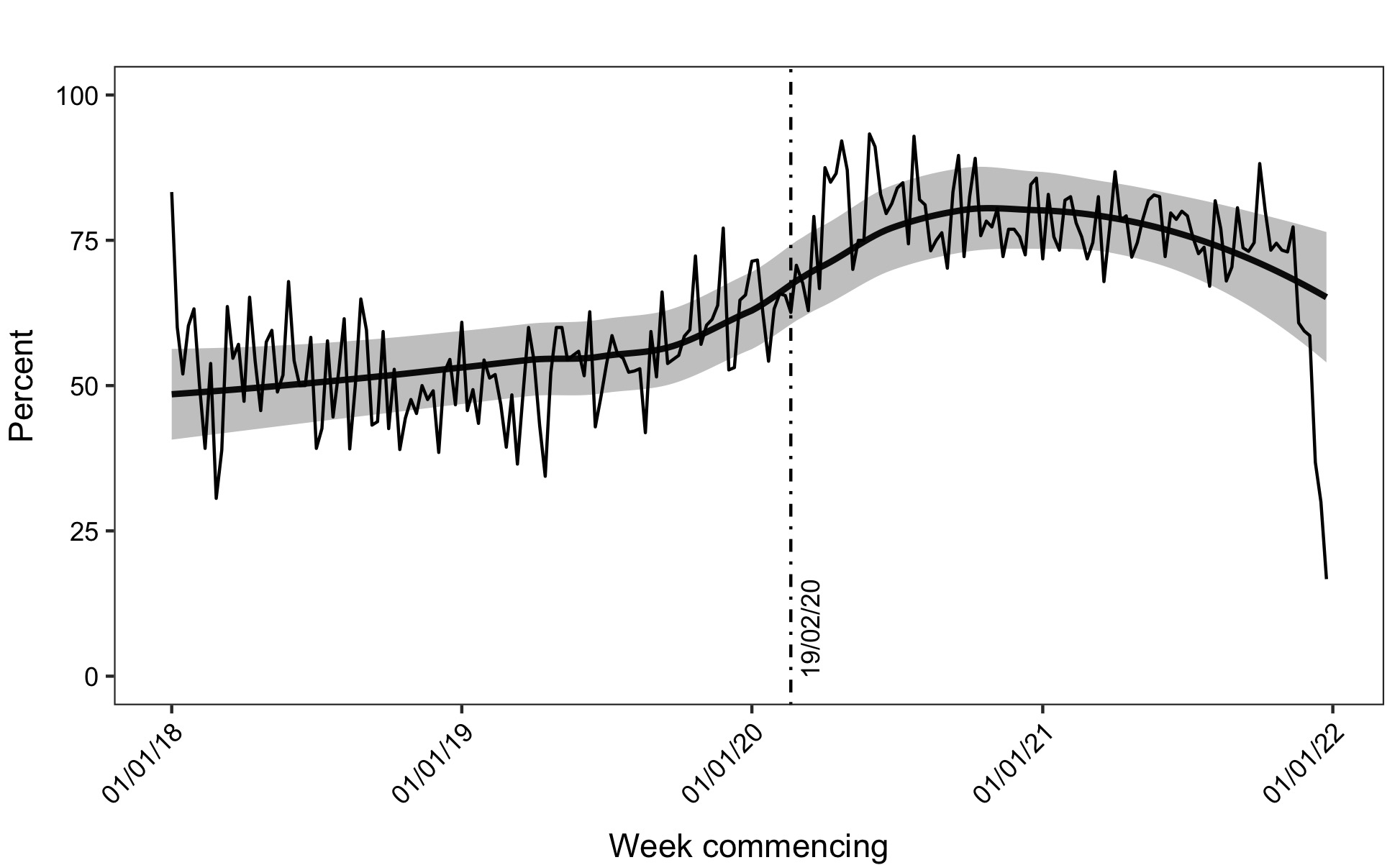   \|  \| IRR \| 95% CI \| SE \| p-value \| \| --- \| --- \| --- \| --- \| --- \| \| Time \| 1.002 \| 1.001-1.003 \| 0.001 \| 0.001 \| \| Level \| 1.215 \| 1.094-1.350 \| 0.054 \| <0.001 \| \| Slope \| 1.003 \| 0.999-1.008 \| 0.002 \| 0.147 \| \| Slope^2^ \| 1.000 \| 1.000-1.000 \| 0.000 \| 0.006 \| \| Slope^3^ \|  \|  \|  \|  \| |
| **Number of 4 week quits** | **4 week quits as a % of quit dates set** |
| 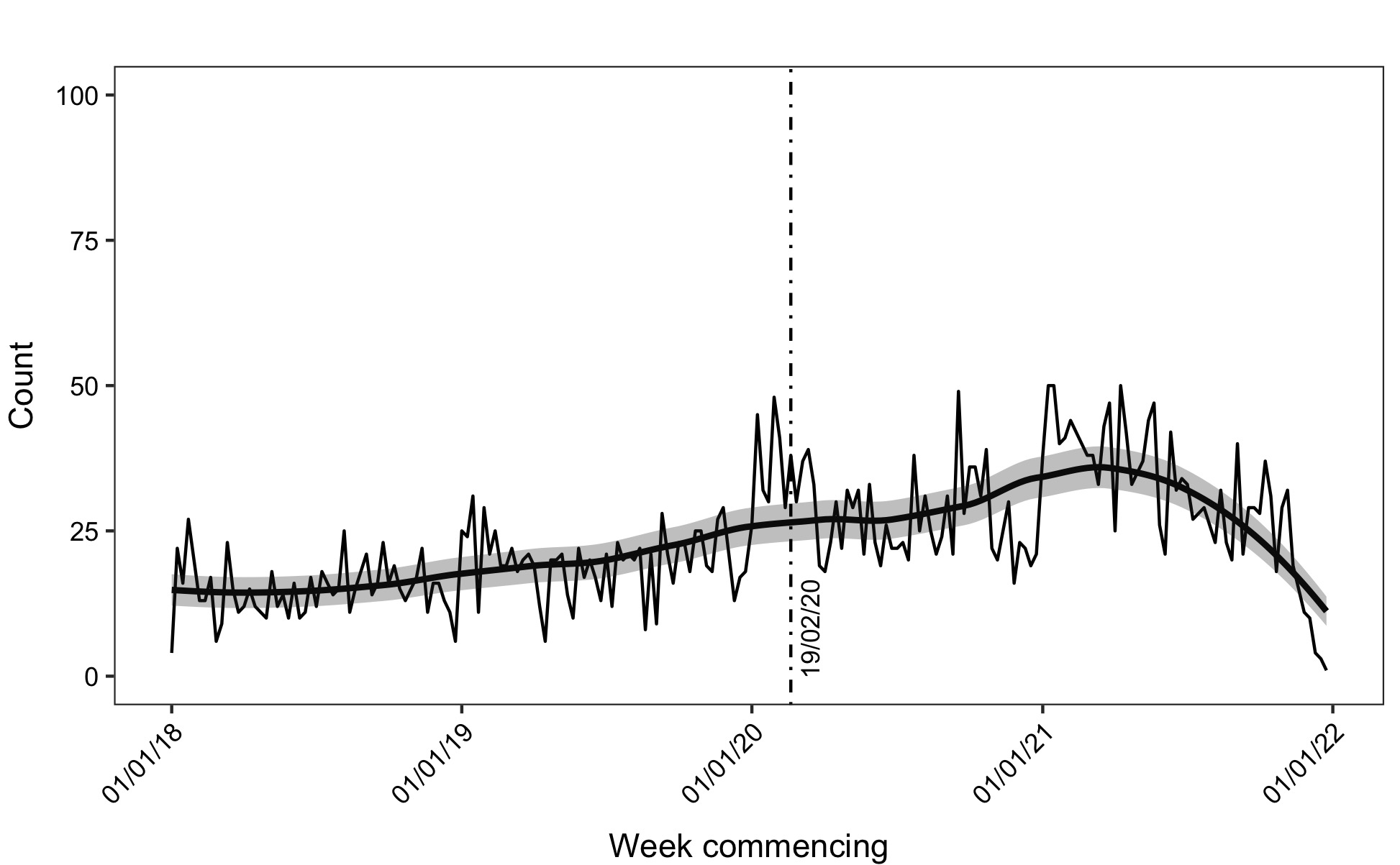   \|  \| IRR \| 95% CI \| SE \| p-value \| \| --- \| --- \| --- \| --- \| --- \| \| Time \| 1.007 \| 1.005-1.008 \| 0.001 \| <0.001 \| \| Level \| 1.321 \| 1.042-1.674 \| 0.122 \| 0.024 \| \| Slope \| 0.964 \| 0.945-0.982 \| 0.010 \| <0.001 \| \| Slope^2^ \| 1.001 \| 1.001-1.002 \| 0.000 \| <0.001 \| \| Slope^3^ \| 1.000 \| 1.000-1.000 \| 0.000 \| <0.001 \| | 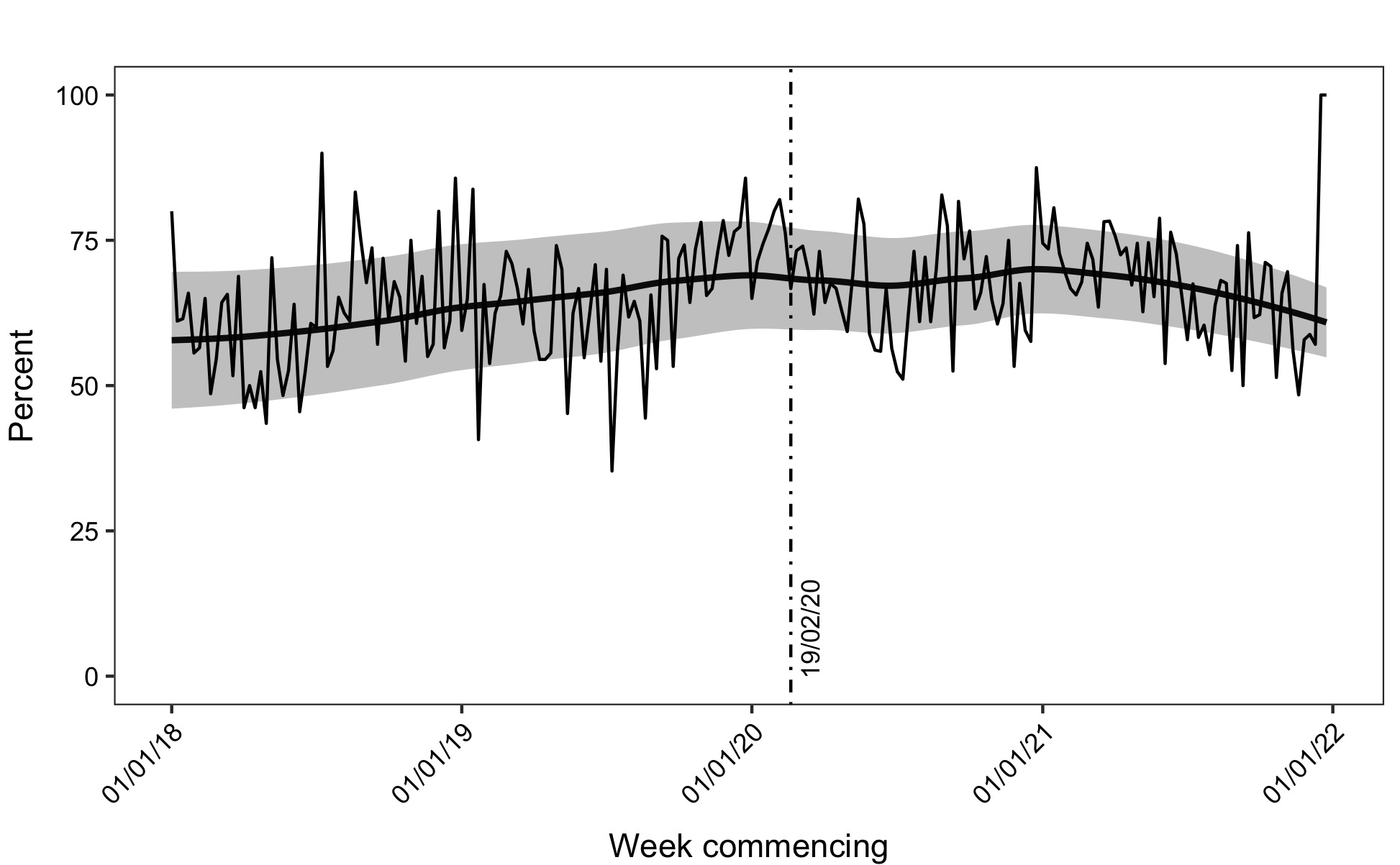   \|  \| IRR \| 95% CI \| SE \| p-value \| \| --- \| --- \| --- \| --- \| --- \| \| Time \| 1.002 \| 1.001-1.003 \| 0.000 \| <0.001 \| \| Level \| 0.904 \| 0.828-0.987 \| 0.045 \| 0.026 \| \| Slope \| 1.002 \| 0.998-1.005 \| 0.002 \| 0.401 \| \| Slope^2^ \| 1.000 \| 1.000-1.000 \| 0.000 \| 0.021 \| \| Slope^3^ \|  \|  \|  \|  \| |

**Supplementary Table 2: Subgroup analyses**

Given the very small numbers, and zero counts in some groups, data for the last 4 weeks of the study period have been excluded from the subgroup analyses to allow model fitting

| **Number of episodes opened** | |
| --- | --- |
| 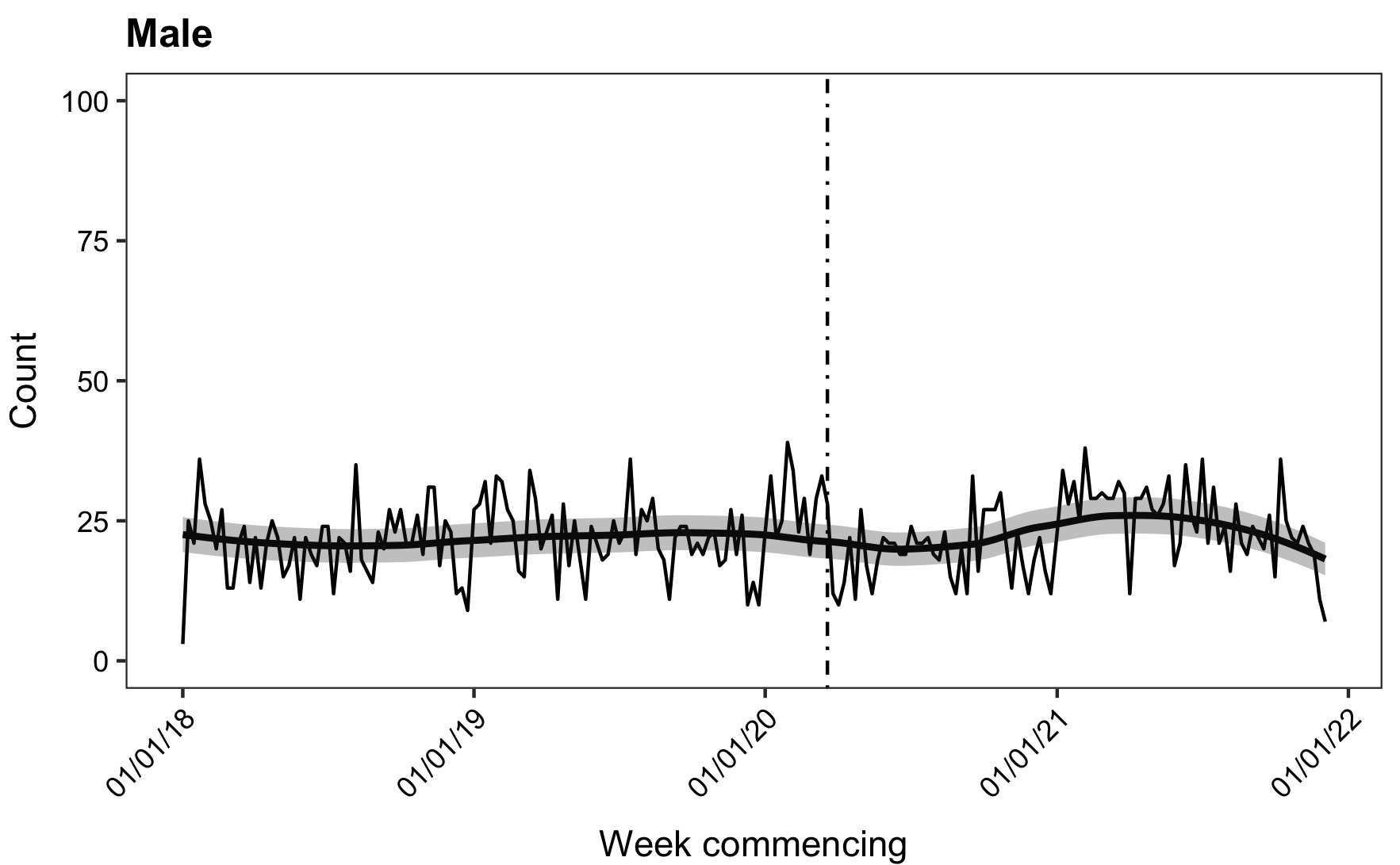   \|  \| IRR \| 95% CI \| SE \| p-value \| \| --- \| --- \| --- \| --- \| --- \| \| Time \| 1.002 \| 1.001-1.003 \| 0.001 \| 0.007 \| \| Level \| 0.773 \| 0.591-1.011 \| 0.138 \| 0.065 \| \| Slope \| 0.995 \| 0.972-1.018 \| 0.012 \| 0.669 \| \| Slope^2^ \| 1.000 \| 1.000-1.001 \| 0.000 \| 0.167 \| \| Slope^3^ \| 1.000 \| 1.000-1.000 \| 0.000 \| 0.049 \| | 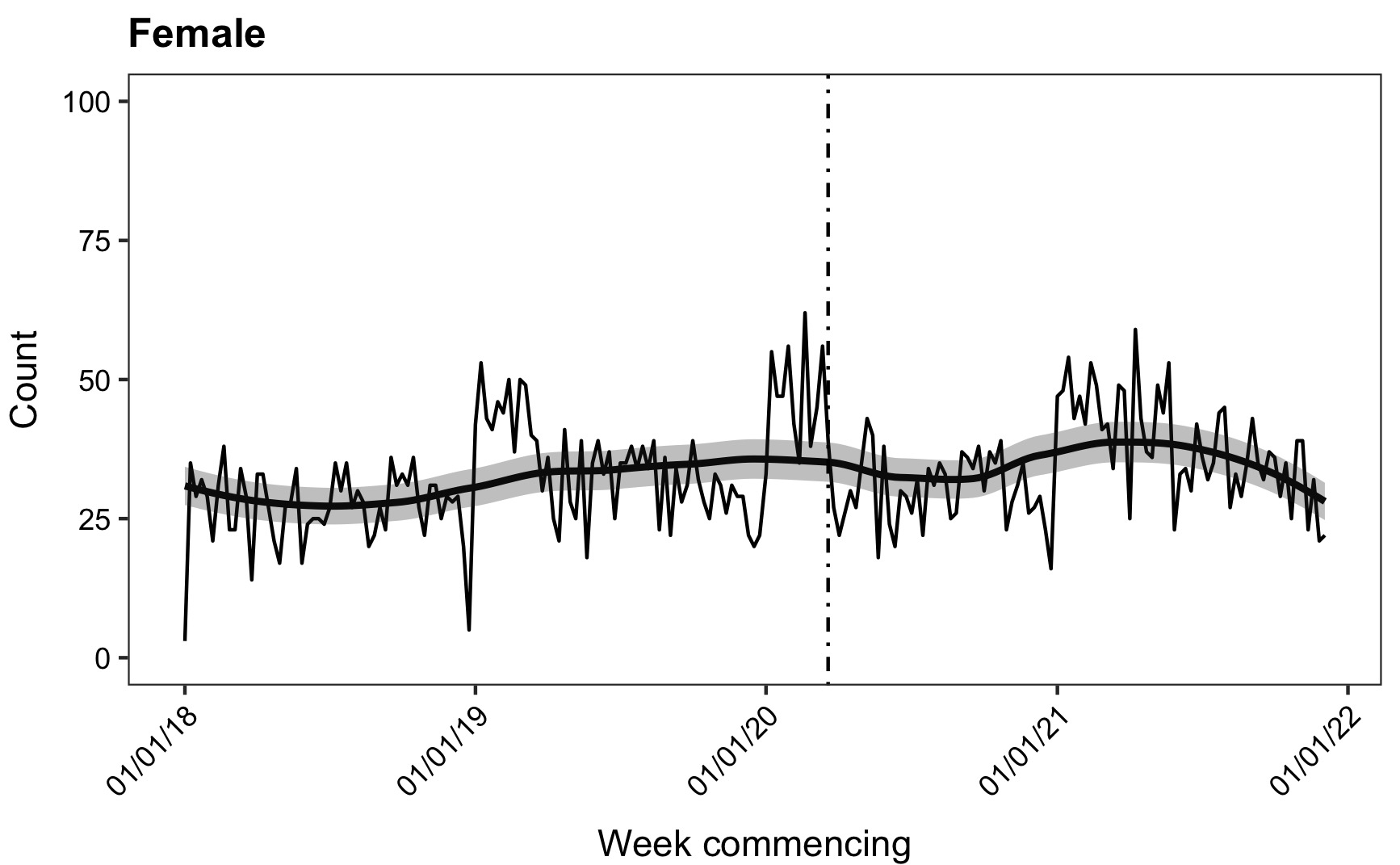   \|  \| IRR \| 95% CI \| SE \| p-value \| \| --- \| --- \| --- \| --- \| --- \| \| Time \| 1.004 \| 1.003-1.005 \| 0.001 \| <0.001 \| \| Level \| 0.666 \| 0.568-0.780 \| 0.082 \| <0.001 \| \| Slope \| 1.009 \| 1.002-1.016 \| 0.004 \| 0.014 \| \| Slope^2^ \| 1.000 \| 1.000-1.000 \| 0.000 \| 0.003 \| \| Slope^3^ \|  \|  \|  \|  \| |
| 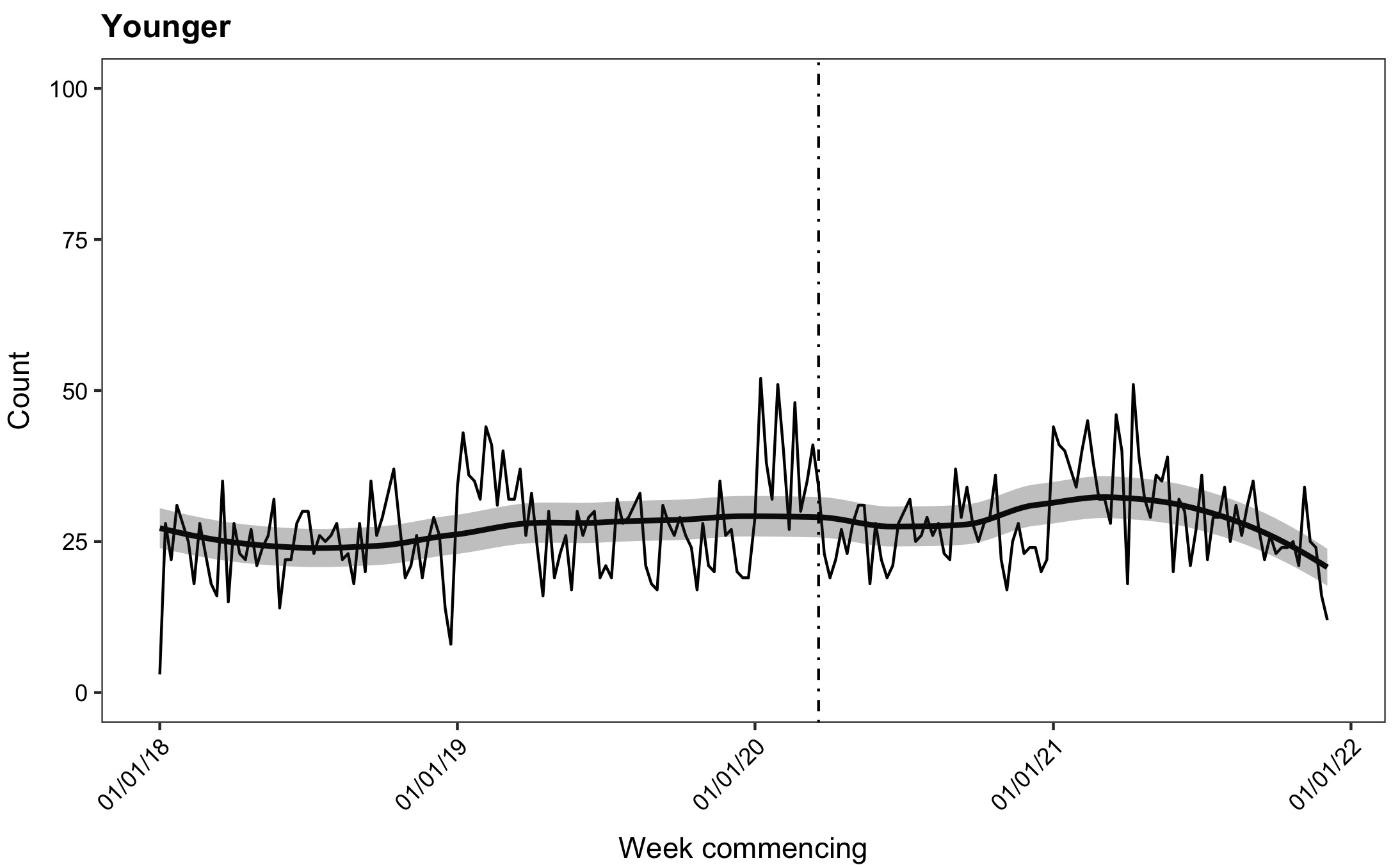   \|  \| IRR \| 95% CI \| SE \| p-value \| \| --- \| --- \| --- \| --- \| --- \| \| Time \| 1.003 \| 1.002-1.005 \| 0.001 \| <0.001 \| \| Level \| 0.705 \| 0.590-0.843 \| 0.092 \| <0.001 \| \| Slope \| 1.011 \| 1.003-1.019 \| 0.004 \| 0.006 \| \| Slope^2^ \| 1.000 \| 1.000-1.000 \| 0.000 \| 0.001 \| \| Slope^3^ \|  \|  \|  \|  \| | 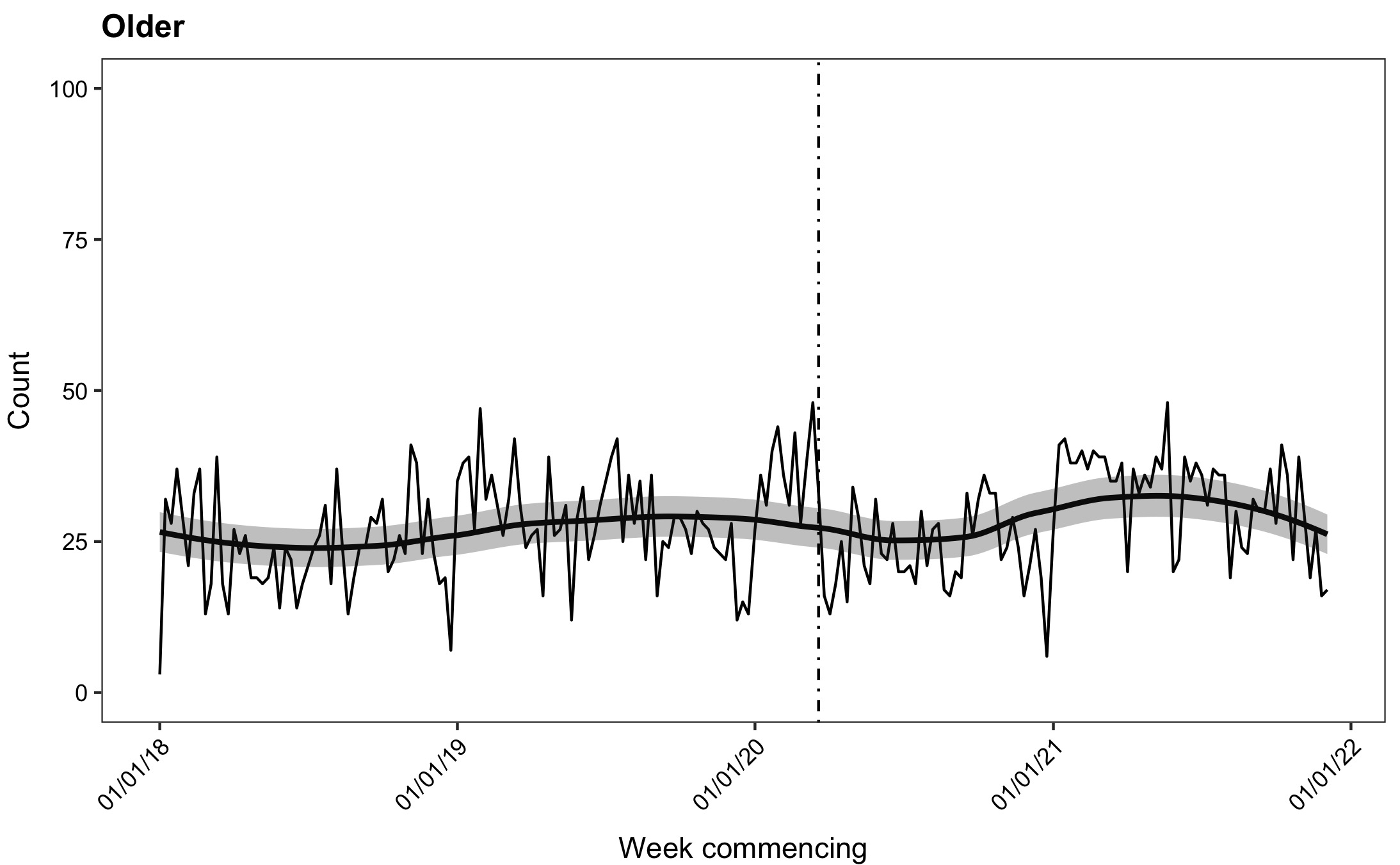   \|  \| IRR \| 95% CI \| SE \| p-value \| \| --- \| --- \| --- \| --- \| --- \| \| Time \| 1.003 \| 1.002-1.005 \| 0.001 \| <0.001 \| \| Level \| 0.593 \| 0.484-0.727 \| 0.105 \| <0.001 \| \| Slope \| 1.014 \| 1.005-1.023 \| 0.005 \| 0.002 \| \| Slope^2^ \| 1.000 \| 1.000-1.000 \| 0.000 \| 0.003 \| \| Slope^3^ \|  \|  \|  \|  \| |
| 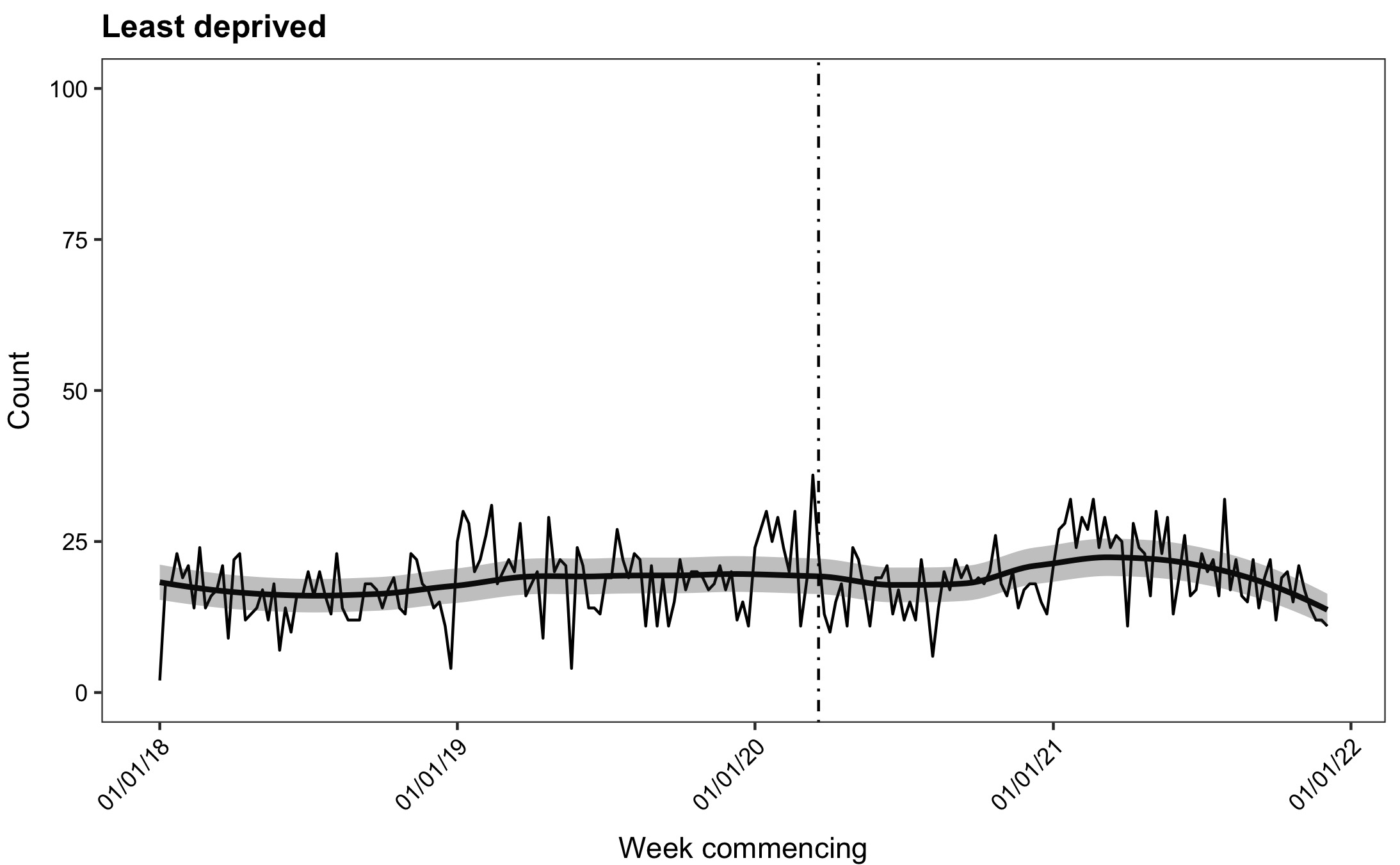   \|  \| IRR \| 95% CI \| SE \| p-value \| \| --- \| --- \| --- \| --- \| --- \| \| Time \| 1.004 \| 1.002-1.005 \| 0.001 \| <0.001 \| \| Level \| 0.620 \| 0.516-0.746 \| 0.095 \| <0.001 \| \| Slope \| 1.015 \| 1.007-1.024 \| 0.004 \| <0.001 \| \| Slope^2^ \| 1.000 \| 1.000-1.000 \| 0.000 \| <0.001 \| \| Slope^3^ \|  \|  \|  \|  \| | 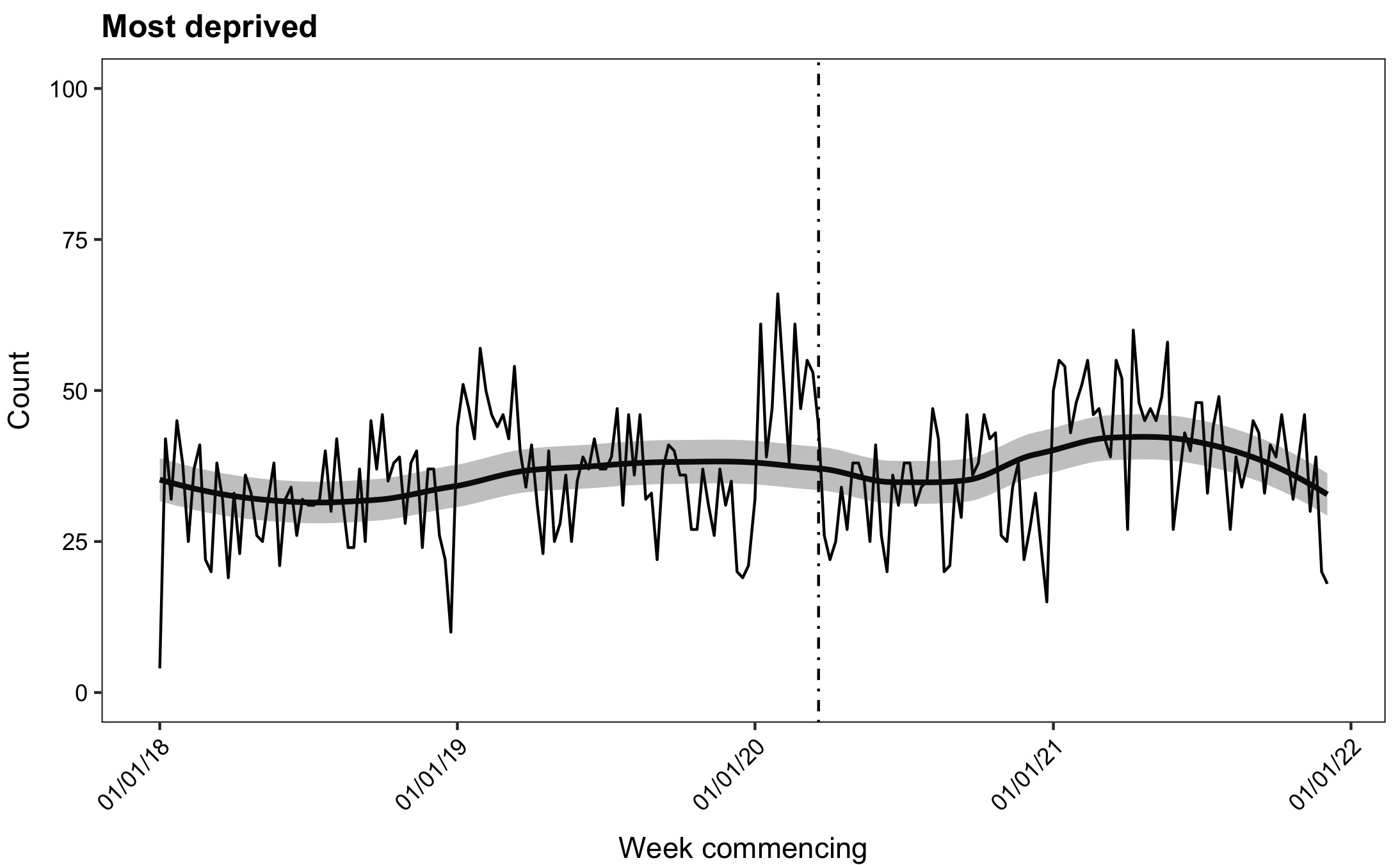   \|  \| IRR \| 95% CI \| SE \| p-value \| \| --- \| --- \| --- \| --- \| --- \| \| Time \| 1.003 \| 1.002-1.005 \| 0.001 \| <0.001 \| \| Level \| 0.659 \| 0.555-0.783 \| 0.088 \| <0.001 \| \| Slope \| 1.011 \| 1.003-1.018 \| 0.004 \| 0.006 \| \| Slope^2^ \| 1.000 \| 1.000-1.000 \| 0.000 \| 0.003 \| \| Slope^3^ \|  \|  \|  \|  \| |

| **Number of quit dates set** | |
| --- | --- |
| 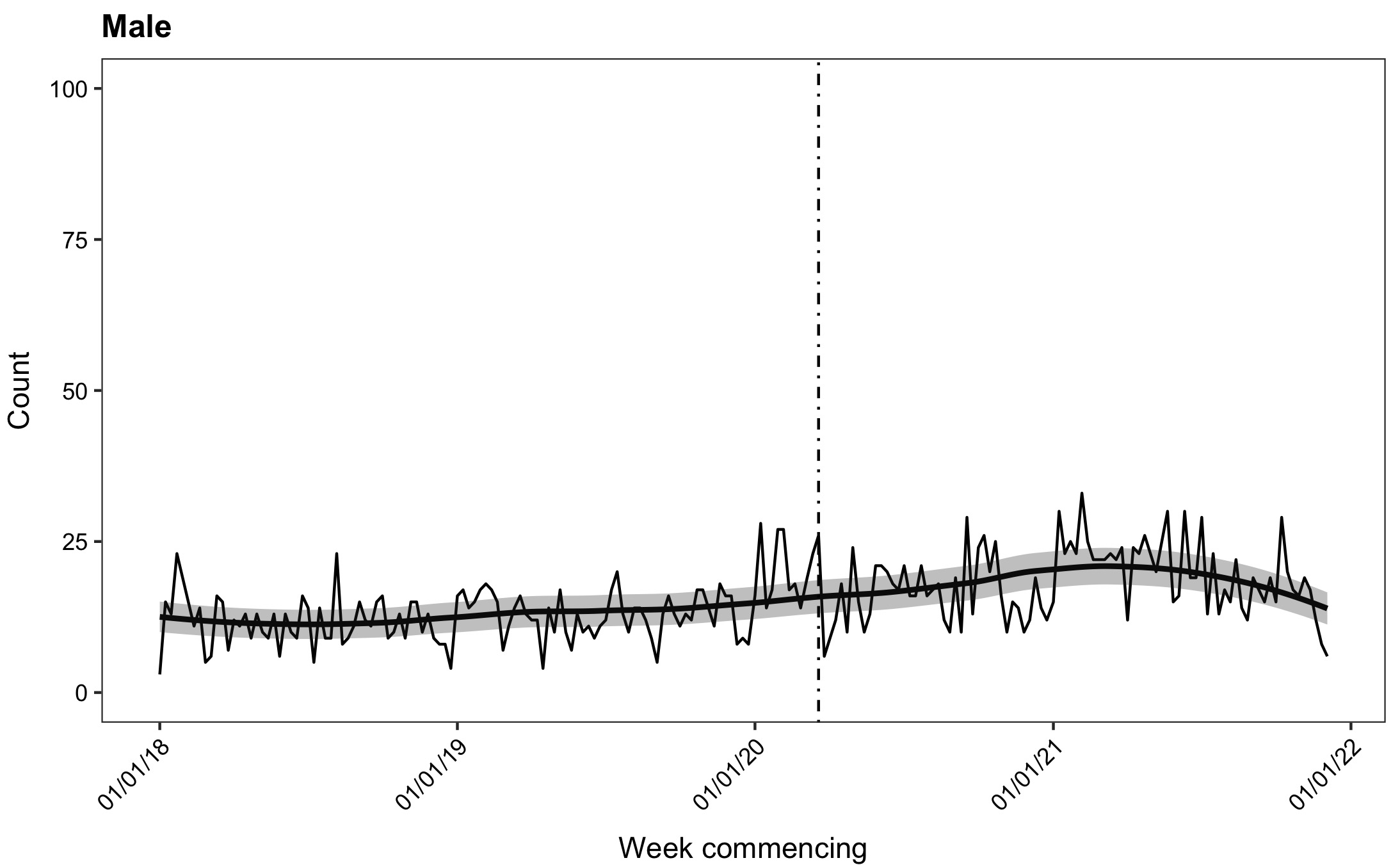   \|  \| IRR \| 95% CI \| SE \| p-value \| \| --- \| --- \| --- \| --- \| --- \| \| Time \| 1.004 \| 1.002-1.005 \| 0.001 \| <0.001 \| \| Level \| 0.877 \| 0.706-1.091 \| 0.112 \| 0.244 \| \| Slope \| 1.013 \| 1.003-1.022 \| 0.005 \| 0.009 \| \| Slope^2^ \| 1.000 \| 1.000-1.000 \| 0.000 \| 0.002 \| \| Slope^3^ \|  \|  \|  \|  \| | 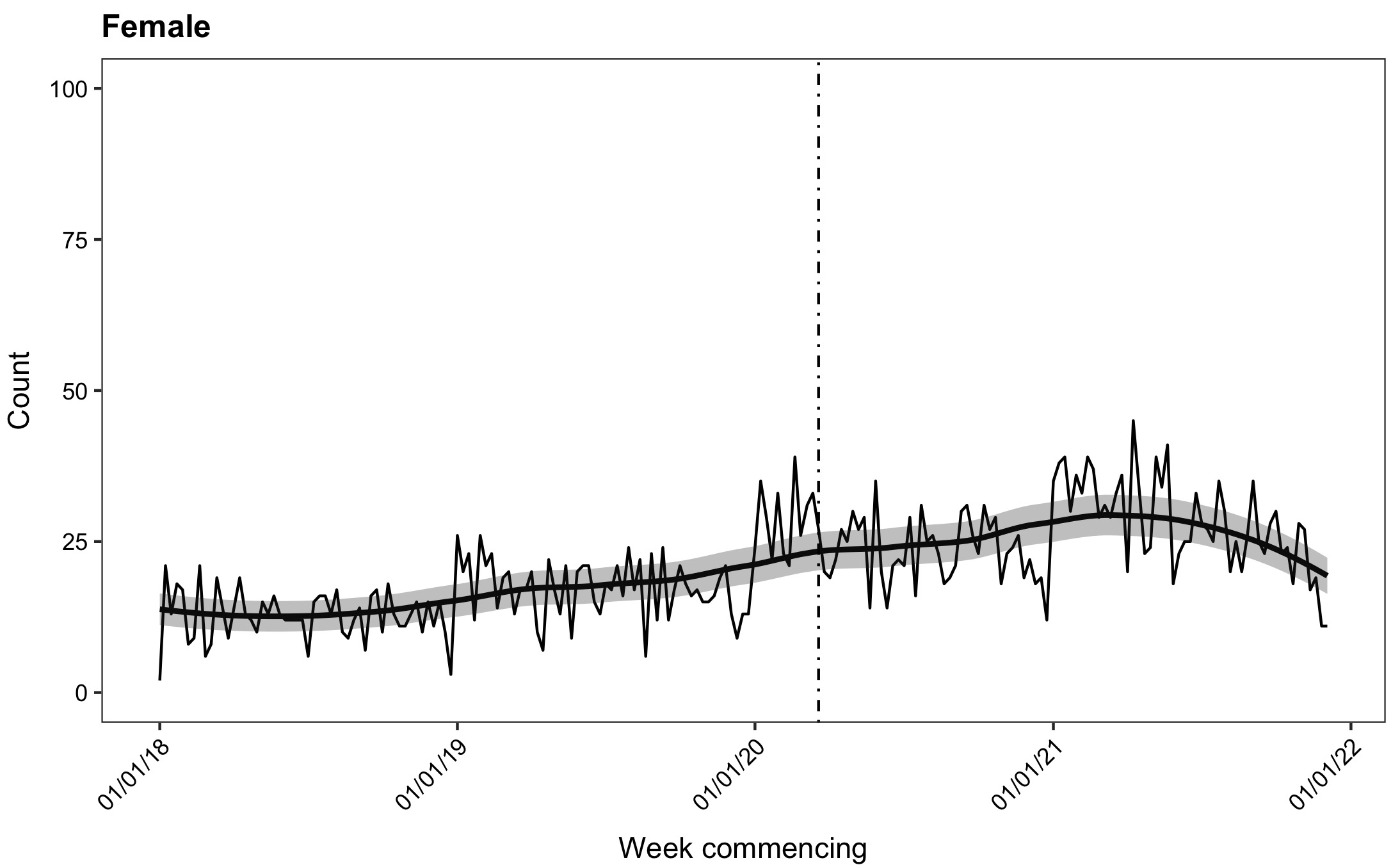   \|  \| IRR \| 95% CI \| SE \| p-value \| \| --- \| --- \| --- \| --- \| --- \| \| Time \| 1.007 \| 1.005-1.008 \| 0.001 \| <0.001 \| \| Level \| 0.894 \| 0.770-1.037 \| 0.077 \| 0.144 \| \| Slope \| 1.006 \| 1.000-1.012 \| 0.003 \| 0.056 \| \| Slope^2^ \| 1.000 \| 1.000-1.000 \| 0.000 \| <0.001 \| \| Slope^3^ \|  \|  \|  \|  \| |
| 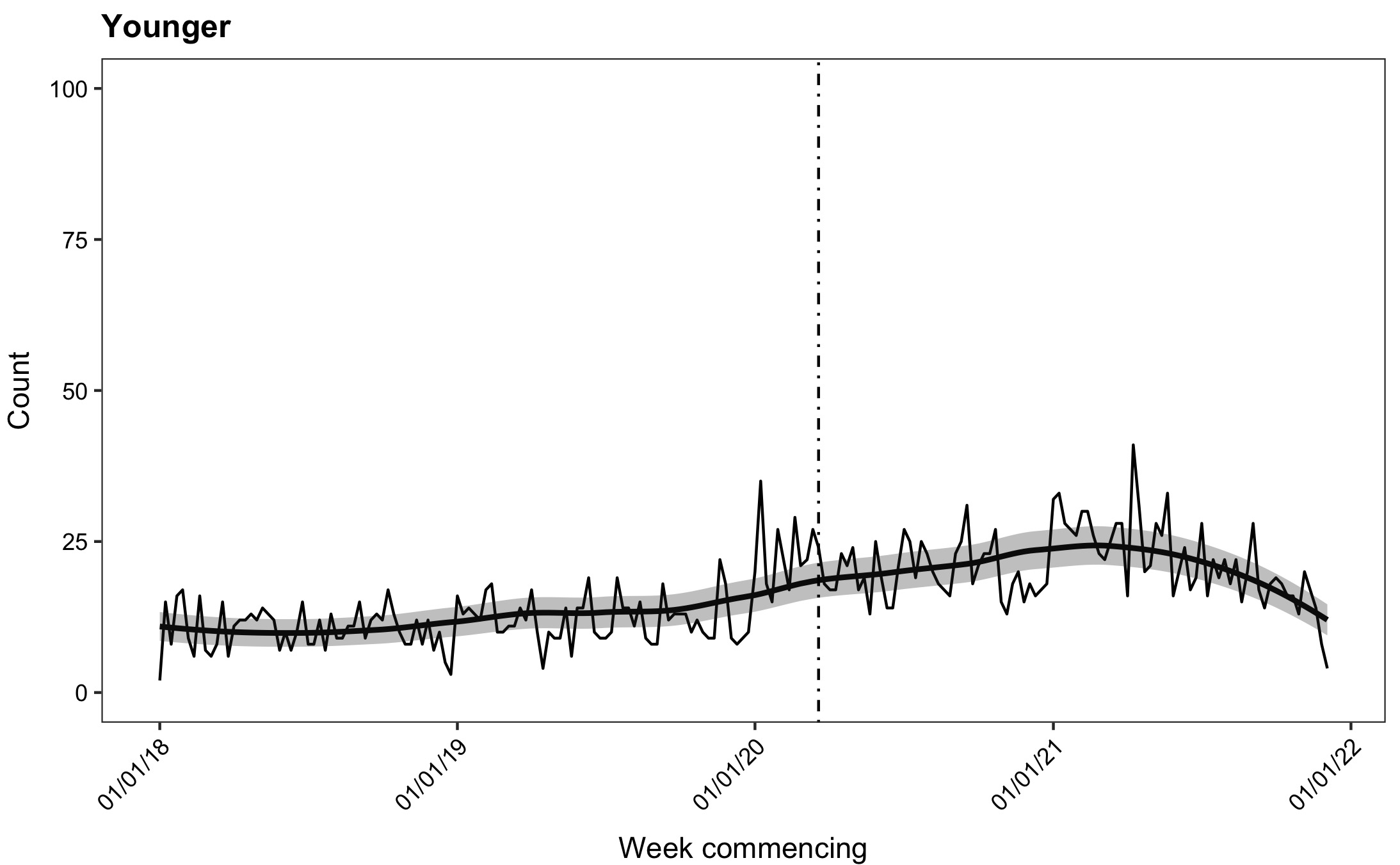   \|  \| IRR \| 95% CI \| SE \| p-value \| \| --- \| --- \| --- \| --- \| --- \| \| Time \| 1.006 \| 1.004-1.008 \| 0.001 \| <0.001 \| \| Level \| 1.000 \| 0.819-1.220 \| 0.103 \| 0.997 \| \| Slope \| 1.010 \| 1.001-1.019 \| 0.004 \| 0.026 \| \| Slope^2^ \| 1.000 \| 1.000-1.000 \| 0.000 \| <0.001 \| \| Slope^3^ \|  \|  \|  \|  \| | 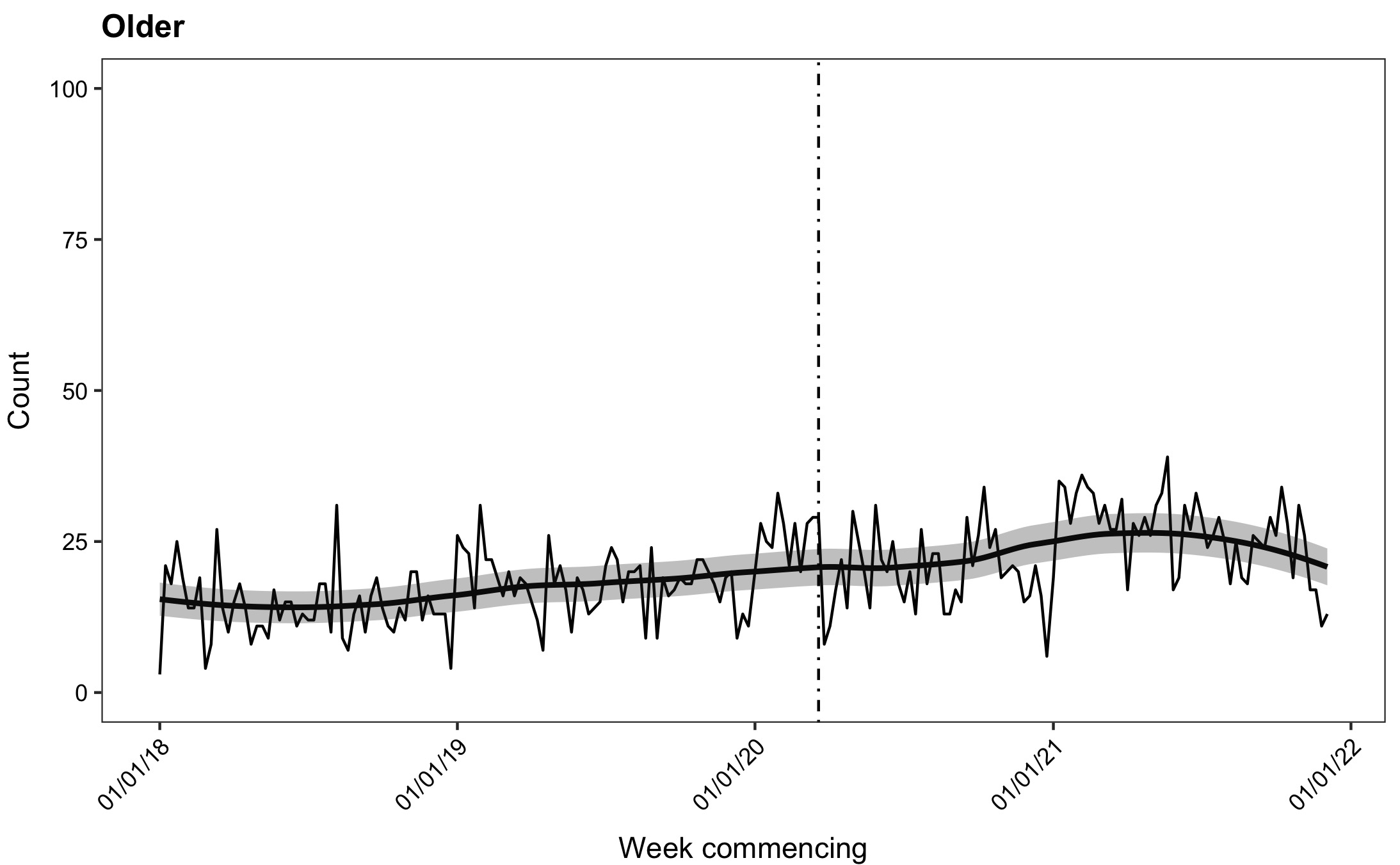   \|  \| IRR \| 95% CI \| SE \| p-value \| \| --- \| --- \| --- \| --- \| --- \| \| Time \| 1.005 \| 1.003-1.006 \| 0.001 \| <0.001 \| \| Level \| 0.782 \| 0.645-0.950 \| 0.100 \| 0.015 \| \| Slope \| 1.009 \| 1.001-1.017 \| 0.004 \| 0.033 \| \| Slope^2^ \| 1.000 \| 1.000-1.000 \| 0.000 \| 0.011 \| \| Slope^3^ \|  \|  \|  \|  \| |
| 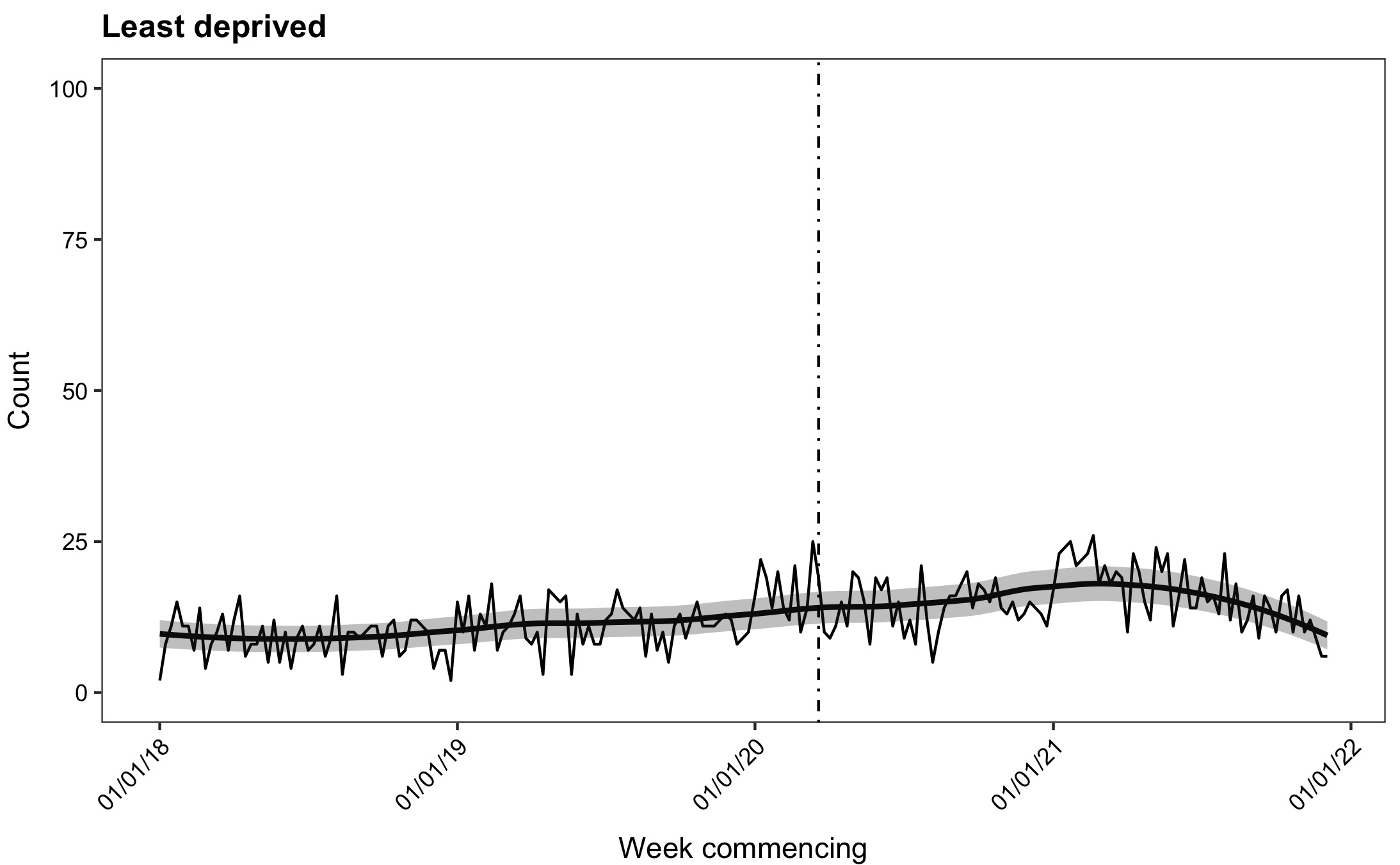   \|  \| IRR \| 95% CI \| SE \| p-value \| \| --- \| --- \| --- \| --- \| --- \| \| Time \| 1.005 \| 1.004-1.007 \| 0.001 \| <0.001 \| \| Level \| 0.809 \| 0.671-0.976 \| 0.097 \| 0.030 \| \| Slope \| 1.014 \| 1.005-1.022 \| 0.004 \| 0.002 \| \| Slope^2^ \| 1.000 \| 1.000-1.000 \| 0.000 \| <0.001 \| \| Slope^3^ \|  \|  \|  \|  \| | 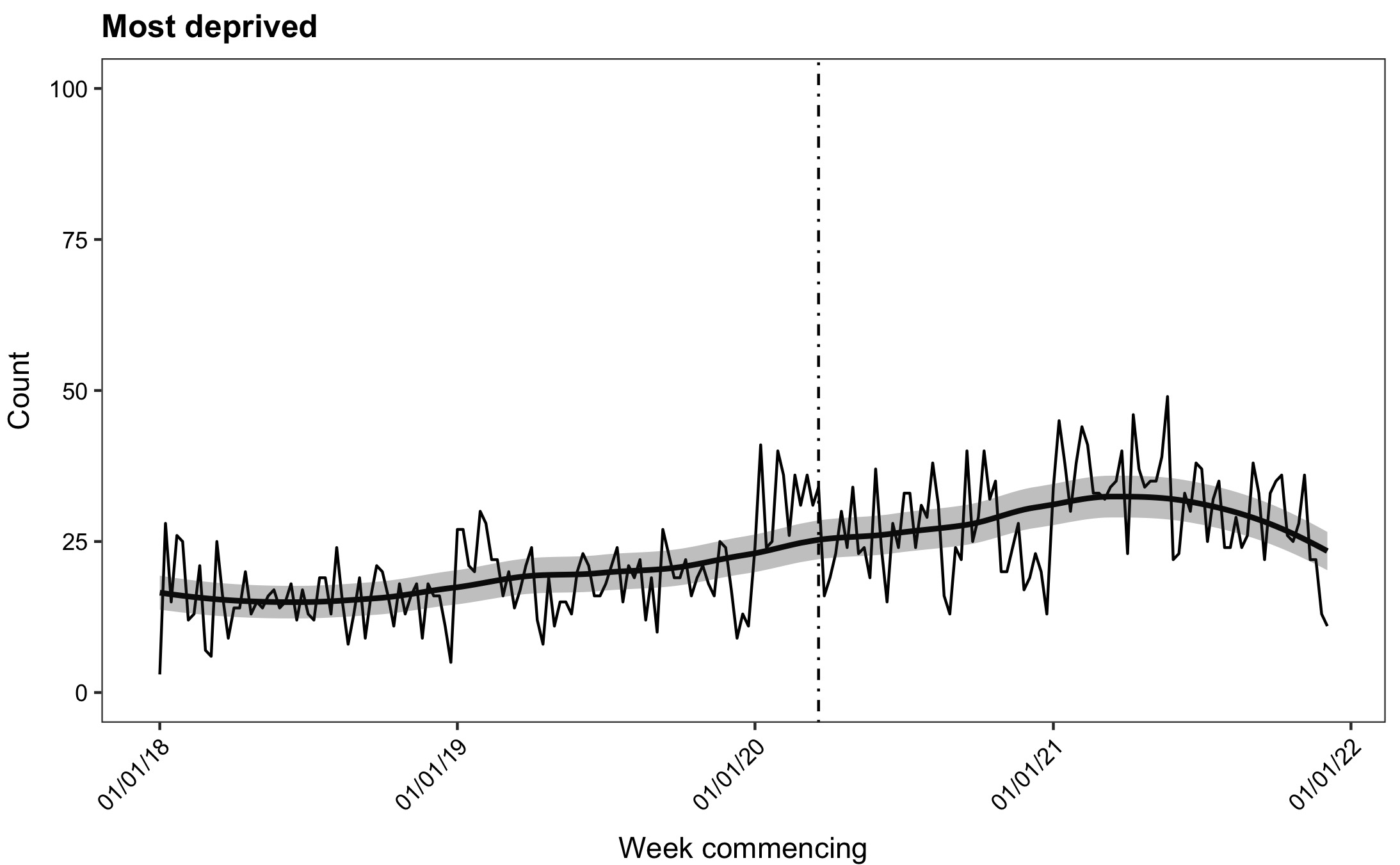   \|  \| IRR \| 95% CI \| SE \| p-value \| \| --- \| --- \| --- \| --- \| --- \| \| Time \| 1.006 \| 1.004-1.007 \| 0.001 \| <0.001 \| \| Level \| 0.927 \| 0.765-1.124 \| 0.099 \| 0.445 \| \| Slope \| 1.006 \| 0.998-1.015 \| 0.004 \| 0.129 \| \| Slope^2^ \| 1.000 \| 1.000-1.000 \| 0.000 \| 0.018 \| \| Slope^3^ \|  \|  \|  \|  \|   c |

| **Number of 4 week quits** | |
| --- | --- |
| 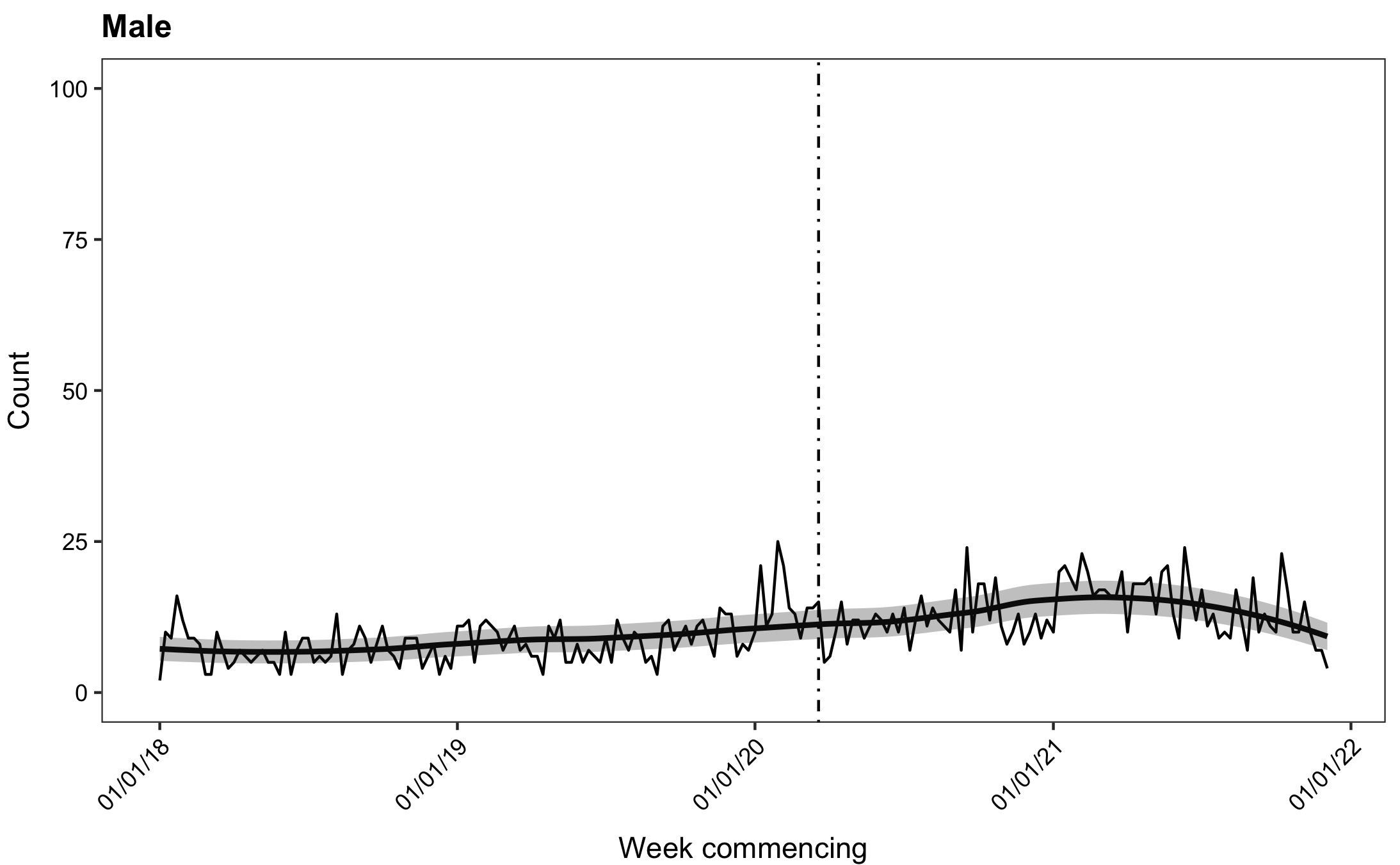   \|  \| IRR \| 95% CI \| SE \| p-value \| \| --- \| --- \| --- \| --- \| --- \| \| Time \| 1.006 \| 1.004-1.008 \| 0.001 \| <0.001 \| \| Level \| 0.814 \| 0.640-1.035 \| 0.124 \| 0.098 \| \| Slope \| 1.015 \| 1.004-1.025 \| 0.005 \| 0.006 \| \| Slope^2^ \| 1.000 \| 1.000-1.000 \| 0.000 \| <0.001 \| \| Slope^3^ \|  \|  \|  \|  \| | 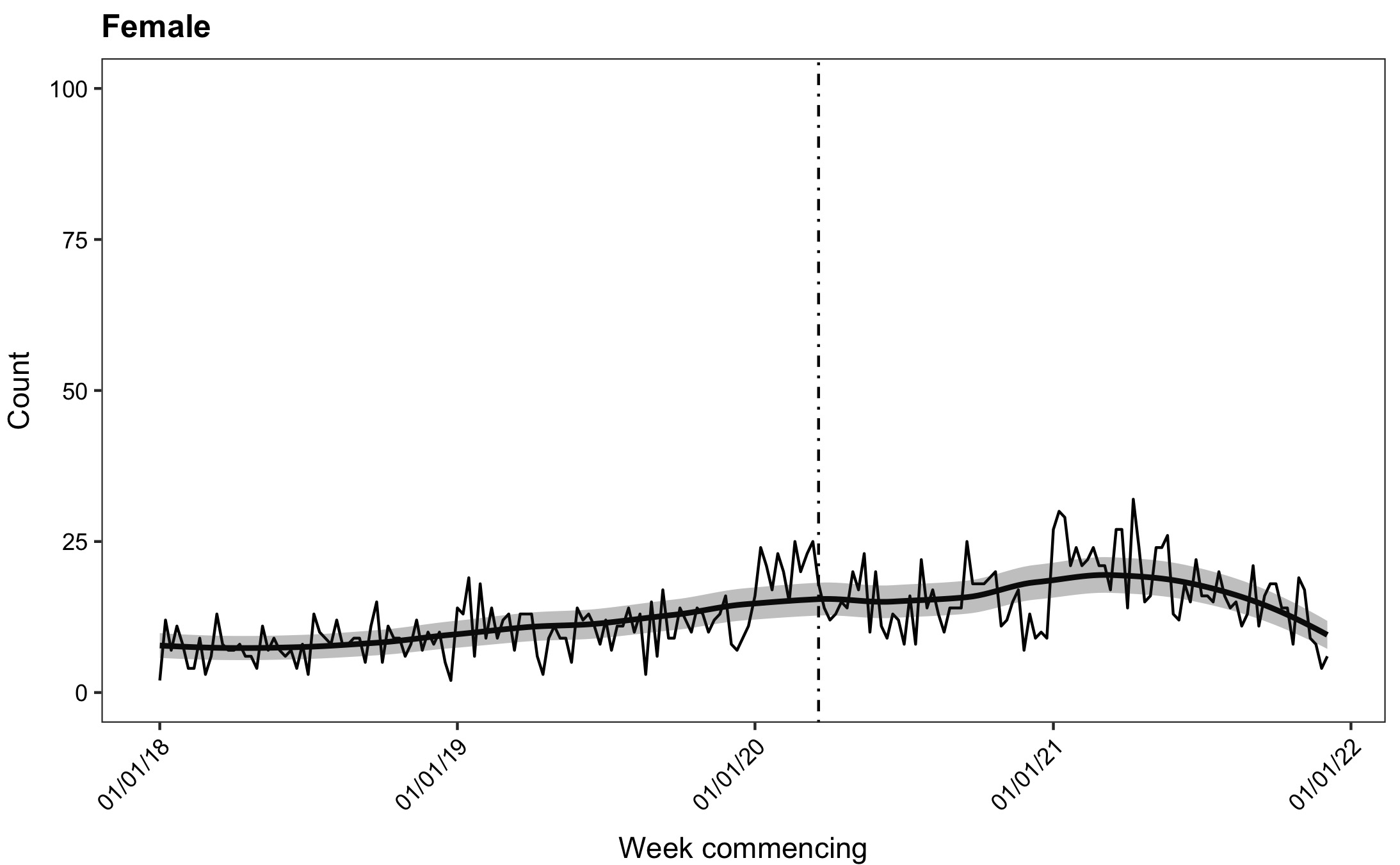   \|  \| IRR \| 95% CI \| SE \| p-value \| \| --- \| --- \| --- \| --- \| --- \| \| Time \| 1.009 \| 1.007-1.010 \| 0.001 \| <0.001 \| \| Level \| 0.936 \| 0.713-1.229 \| 0.140 \| 0.638 \| \| Slope \| 0.984 \| 0.960-1.007 \| 0.012 \| 0.183 \| \| Slope^2^ \| 1.001 \| 1.000-1.001 \| 0.000 \| 0.102 \| \| Slope^3^ \| 1.000 \| 1.000-1.000 \| 0.000 \| 0.025 \| |
| 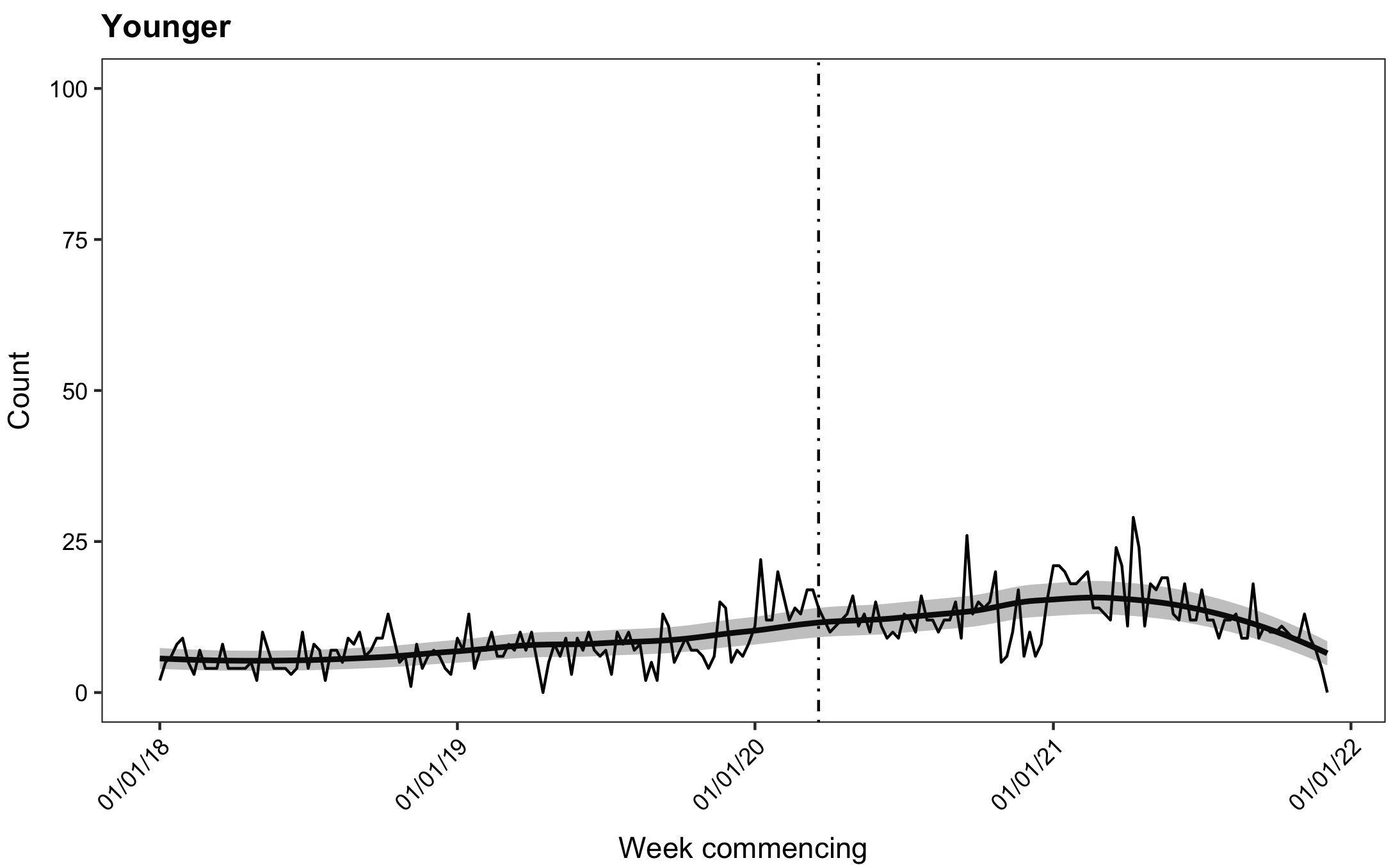   \|  \| IRR \| 95% CI \| SE \| p-value \| \| --- \| --- \| --- \| --- \| --- \| \| Time \| 1.008 \| 1.006-1.011 \| 0.001 \| <0.001 \| \| Level \| 0.841 \| 0.647-1.095 \| 0.136 \| 0.204 \| \| Slope \| 1.014 \| 1.002-1.026 \| 0.006 \| 0.020 \| \| Slope^2^ \| 1.000 \| 1.000-1.000 \| 0.000 \| <0.001 \| \| Slope^3^ \|  \|  \|  \|  \| | 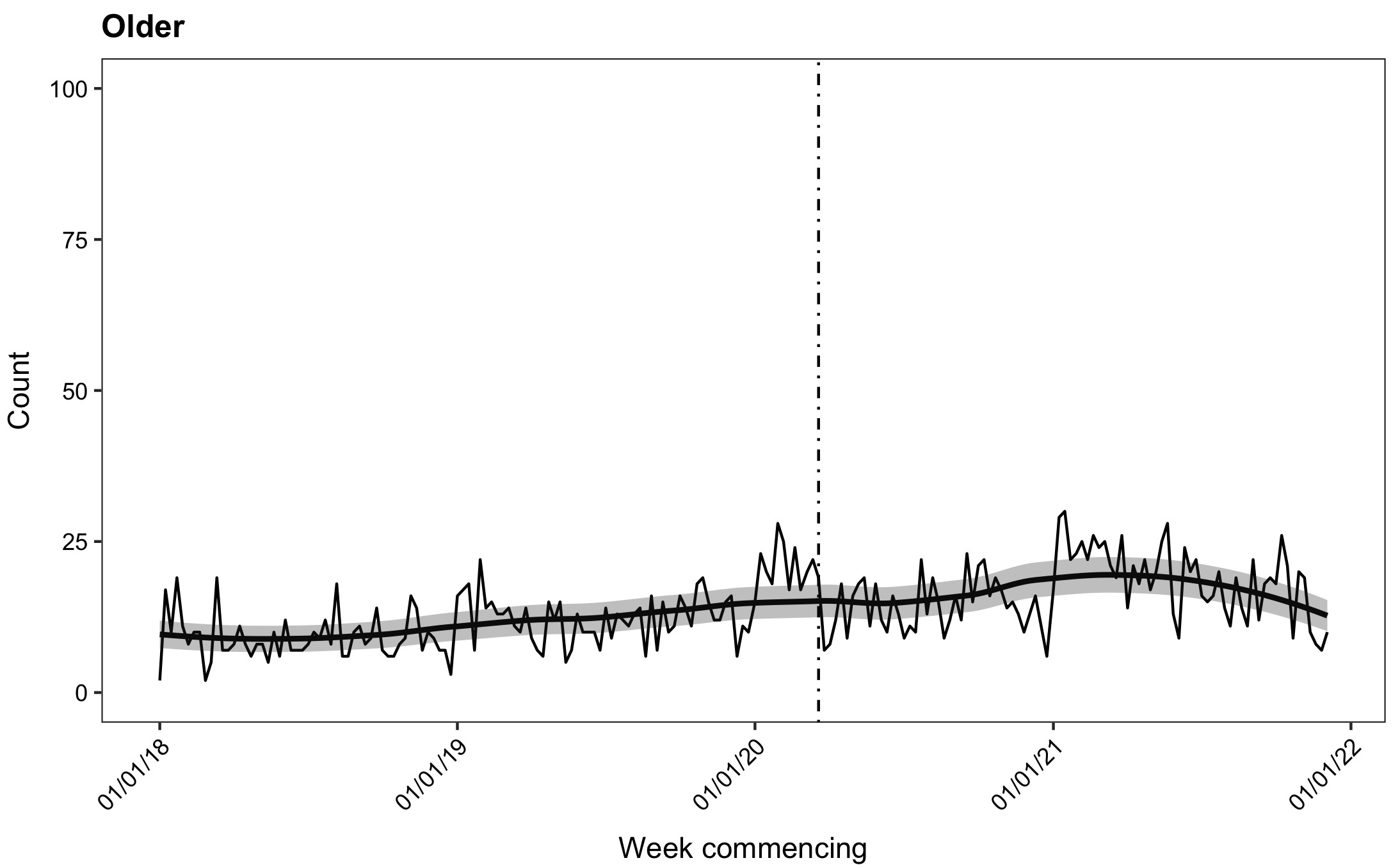   \|  \| IRR \| 95% CI \| SE \| p-value \| \| --- \| --- \| --- \| --- \| --- \| \| Time \| 1.007 \| 1.005-1.009 \| 0.001 \| <0.001 \| \| Level \| 0.717 \| 0.584-0.880 \| 0.106 \| 0.002 \| \| Slope \| 1.011 \| 1.003-1.020 \| 0.004 \| 0.011 \| \| Slope^2^ \| 1.000 \| 1.000-1.000 \| 0.000 \| <0.001 \| \| Slope^3^ \|  \|  \|  \|  \| |
| 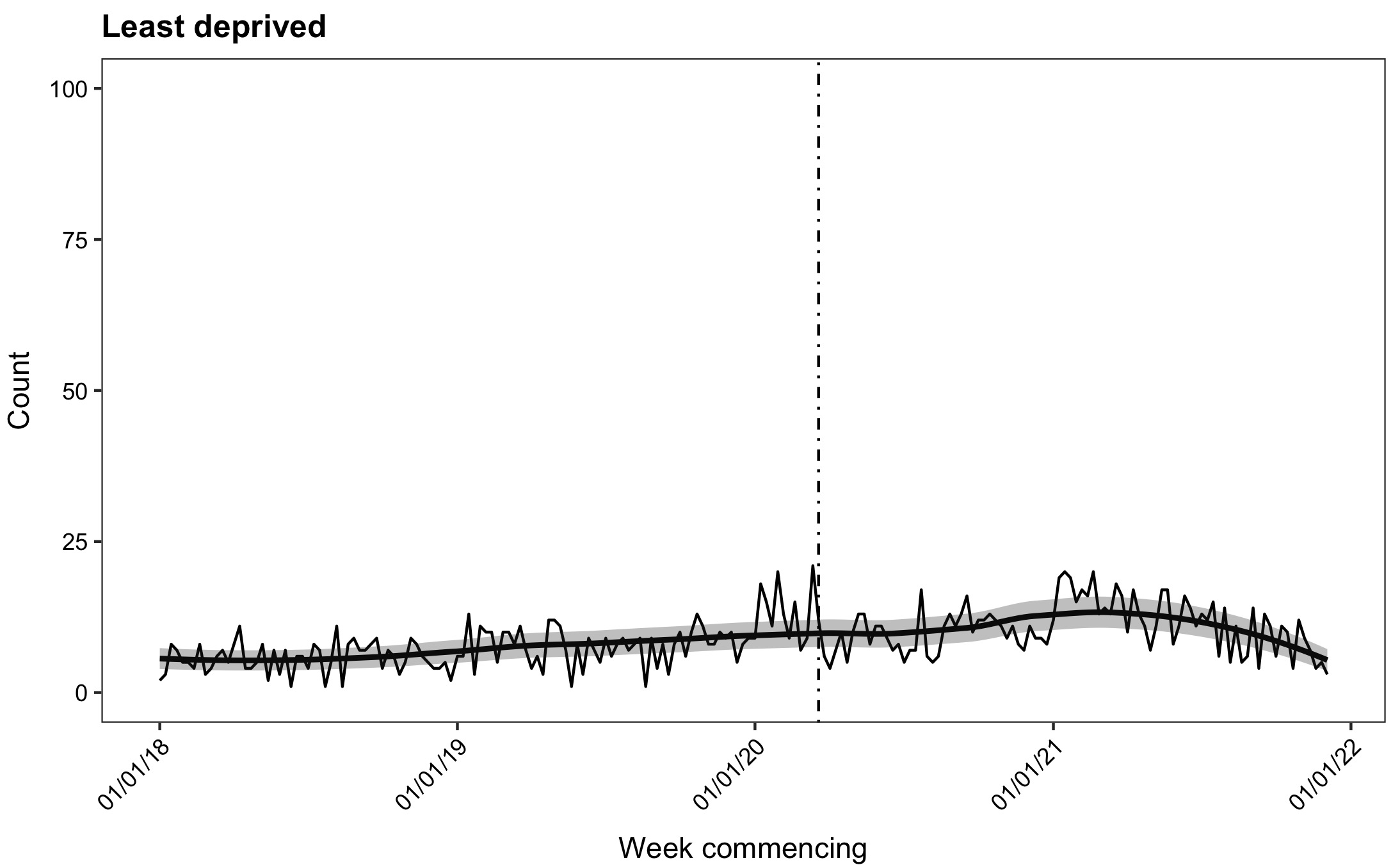   \|  \| IRR \| 95% CI \| SE \| p-value \| \| --- \| --- \| --- \| --- \| --- \| \| Time \| 1.008 \| 1.006-1.010 \| 0.001 \| <0.001 \| \| Level \| 0.602 \| 0.472-0.768 \| 0.125 \| <0.001 \| \| Slope \| 1.022 \| 1.011-1.032 \| 0.005 \| <0.001 \| \| Slope^2^ \| 1.000 \| 1.000-1.000 \| 0.000 \| <0.001 \| \| Slope^3^ \|  \|  \|  \|  \| | 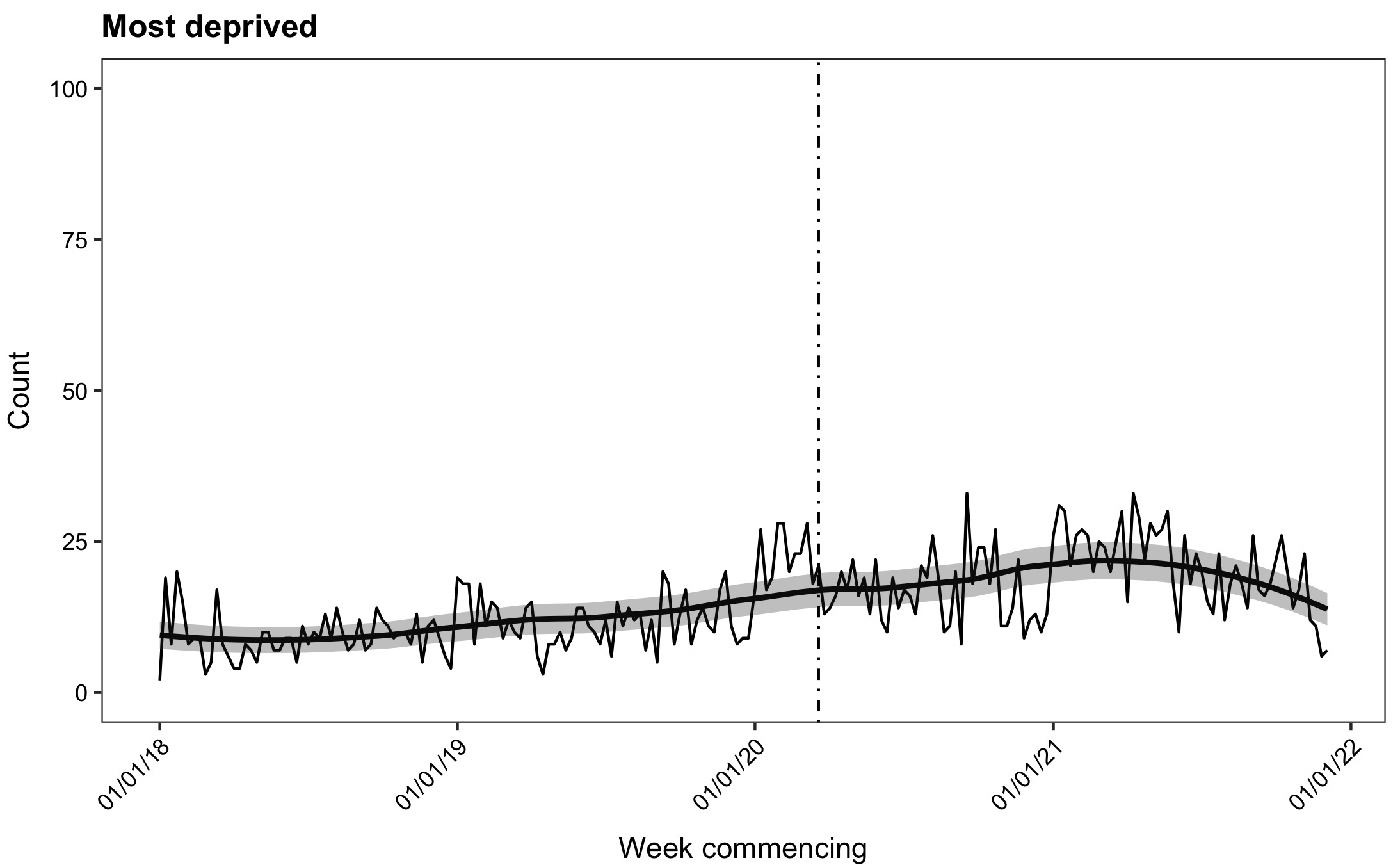   \|  \| IRR \| 95% CI \| SE \| p-value \| \| --- \| --- \| --- \| --- \| --- \| \| Time \| 1.007 \| 1.005-1.009 \| 0.001 \| <0.001 \| \| Level \| 0.881 \| 0.701-1.106 \| 0.117 \| 0.279 \| \| Slope \| 1.007 \| 0.997-1.017 \| 0.005 \| 0.162 \| \| Slope^2^ \| 1.000 \| 1.000-1.000 \| 0.000 \| 0.009 \| \| Slope^3^ \|  \|  \|  \|  \| |

| **Quit dates set as a percentage of episodes opened** | |
| --- | --- |
| 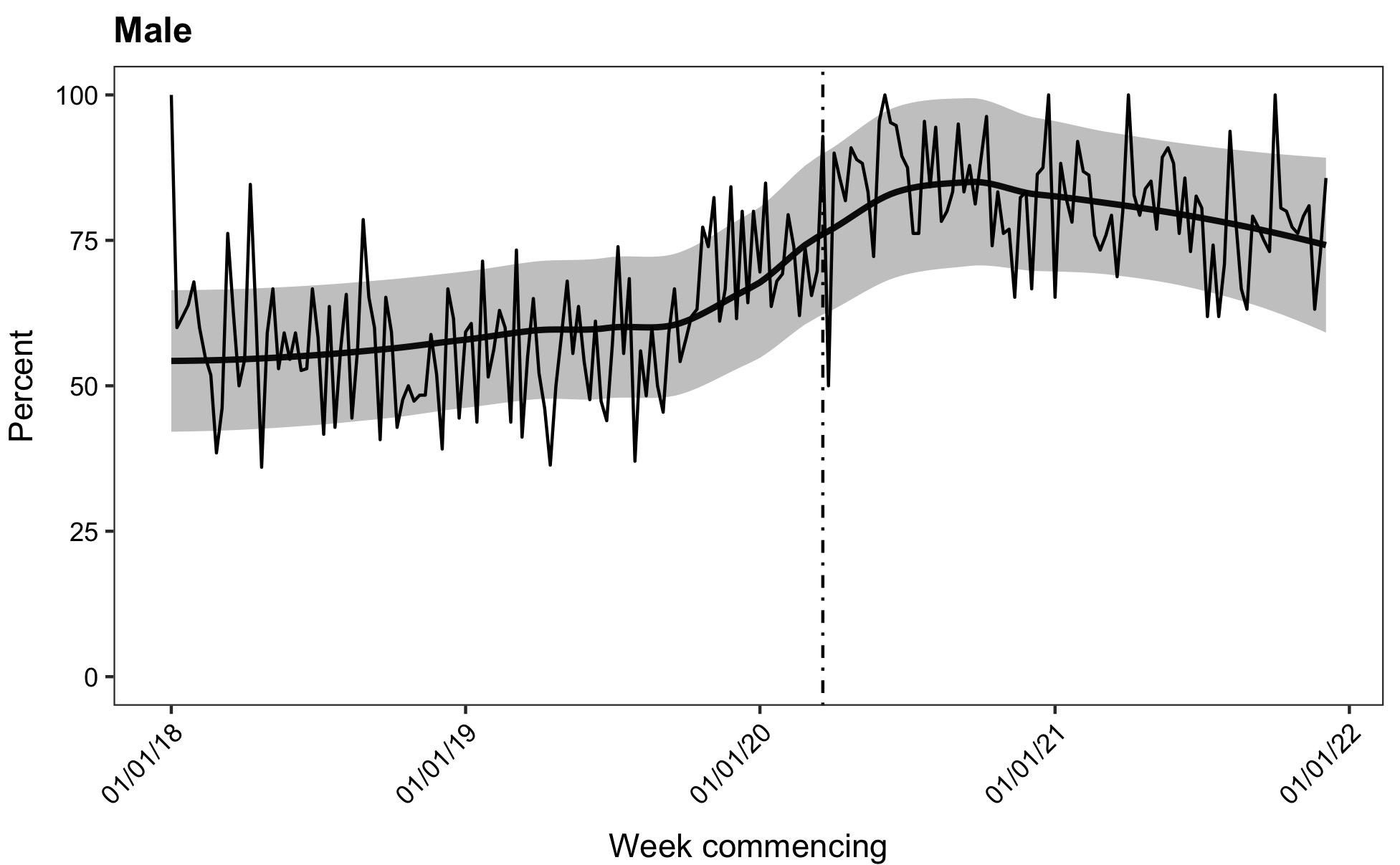   \|  \| IRR \| 95% CI \| SE \| p-value \| \| --- \| --- \| --- \| --- \| --- \| \| Time \| 1.002 \| 1.001-1.003 \| 0.000 \| <0.001 \| \| Level \| 1.356 \| 1.242-1.480 \| 0.045 \| <0.001 \| \| Slope \| 0.997 \| 0.995-0.998 \| 0.001 \| <0.001 \| \| Slope^2^ \|  \|  \|  \|  \| \| Slope^3^ \|  \|  \|  \|  \| | 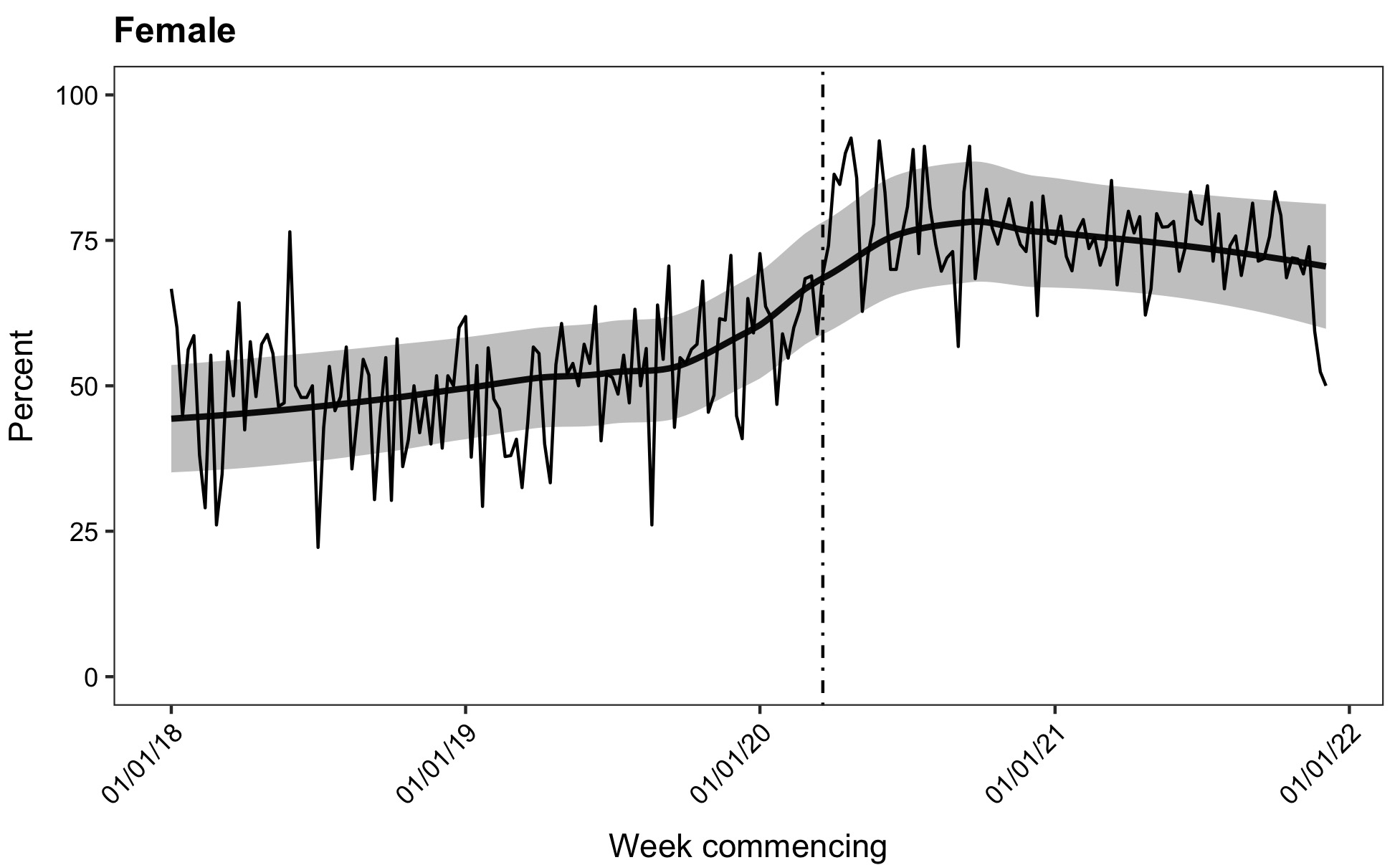   \|  \| IRR \| 95% CI \| SE \| p-value \| \| --- \| --- \| --- \| --- \| --- \| \| Time \| 1.003 \| 1.002-1.003 \| 0.000 \| <0.001 \| \| Level \| 1.373 \| 1.266-1.489 \| 0.042 \| <0.001 \| \| Slope \| 0.996 \| 0.995-0.998 \| 0.001 \| <0.001 \| \| Slope^2^ \|  \|  \|  \|  \| \| Slope^3^ \|  \|  \|  \|  \| |
| 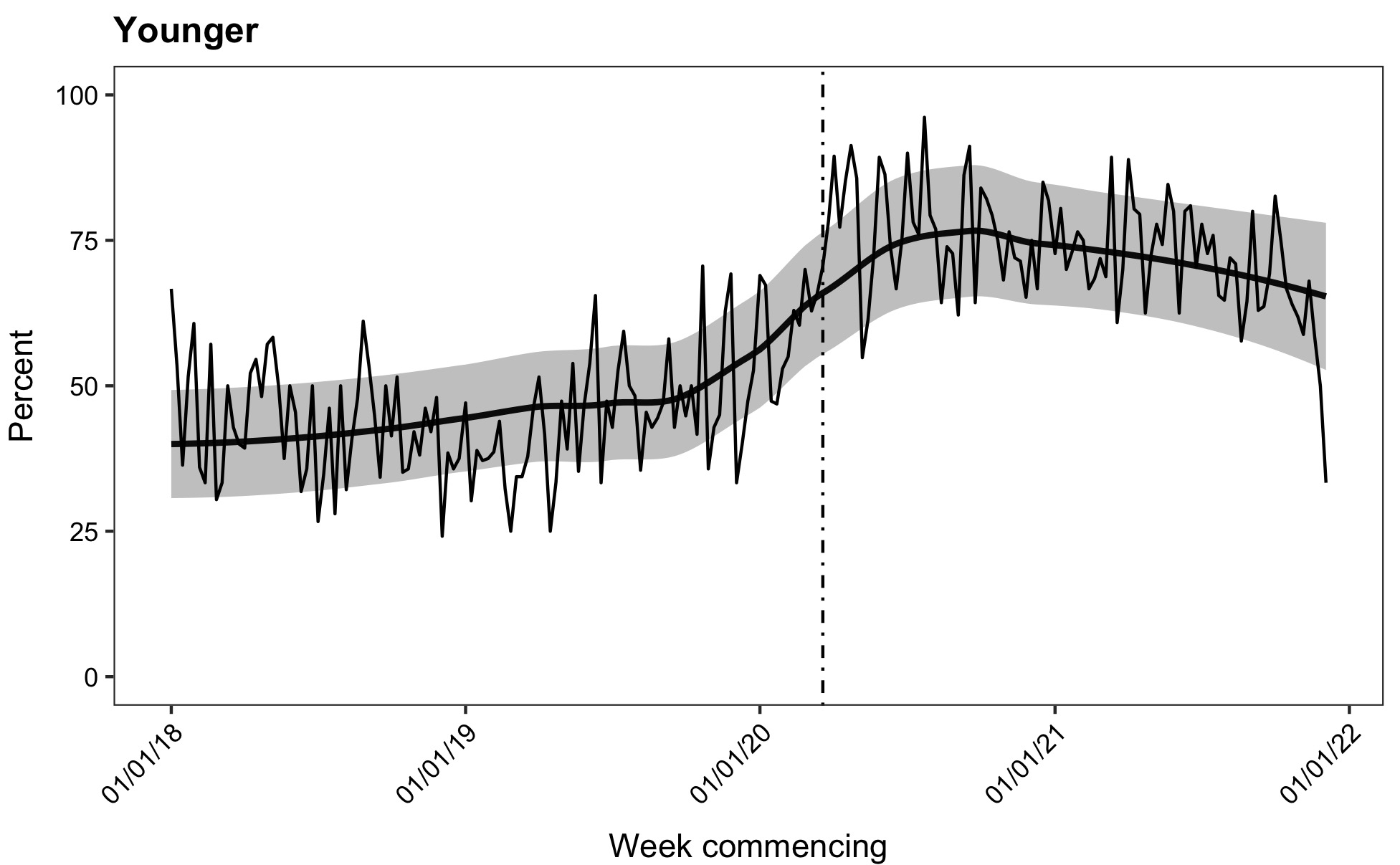   \|  \| IRR \| 95% CI \| SE \| p-value \| \| --- \| --- \| --- \| --- \| --- \| \| Time \| 1.003 \| 1.001-1.004 \| 0.001 \| <0.001 \| \| Level \| 1.498 \| 1.342-1.673 \| 0.057 \| <0.001 \| \| Slope \| 0.995 \| 0.993-0.998 \| 0.001 \| <0.001 \| \| Slope^2^ \|  \|  \|  \|  \| \| Slope^3^ \|  \|  \|  \|  \| | 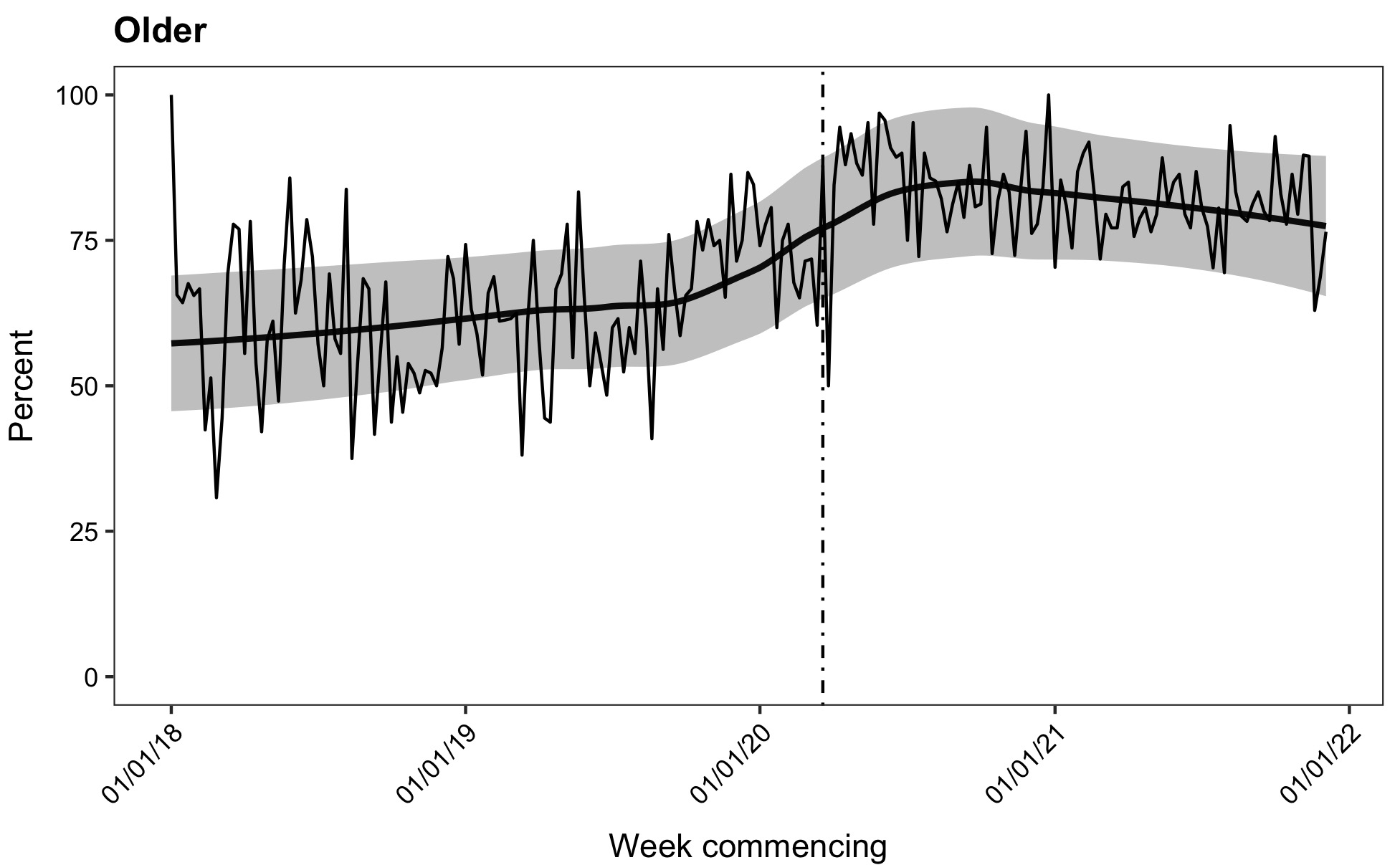   \|  \| IRR \| 95% CI \| SE \| p-value \| \| --- \| --- \| --- \| --- \| --- \| \| Time \| 1.002 \| 1.001-1.003 \| 0.000 \| 0.001 \| \| Level \| 1.275 \| 1.169-1.390 \| 0.044 \| <0.001 \| \| Slope \| 0.997 \| 0.996-0.999 \| 0.001 \| 0.001 \| \| Slope^2^ \|  \|  \|  \|  \| \| Slope^3^ \|  \|  \|  \|  \| |
| 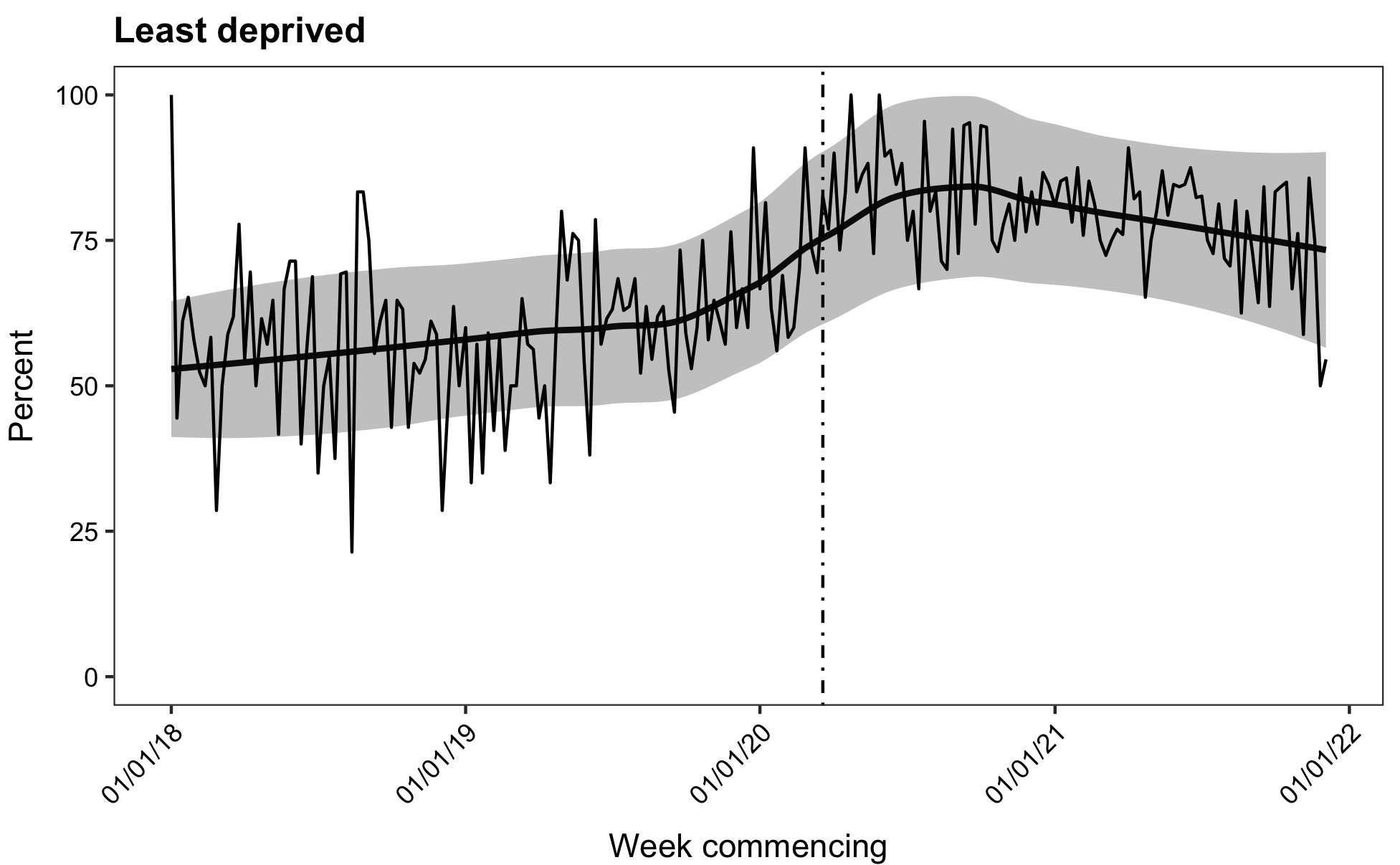   \|  \| IRR \| 95% CI \| SE \| p-value \| \| --- \| --- \| --- \| --- \| --- \| \| Time \| 1.002 \| 1.001-1.003 \| 0.000 \| <0.001 \| \| Level \| 1.343 \| 1.236-1.460 \| 0.043 \| <0.001 \| \| Slope \| 0.996 \| 0.995-0.998 \| 0.001 \| <0.001 \| \| Slope^2^ \|  \|  \|  \|  \| \| Slope^3^ \|  \|  \|  \|  \| | 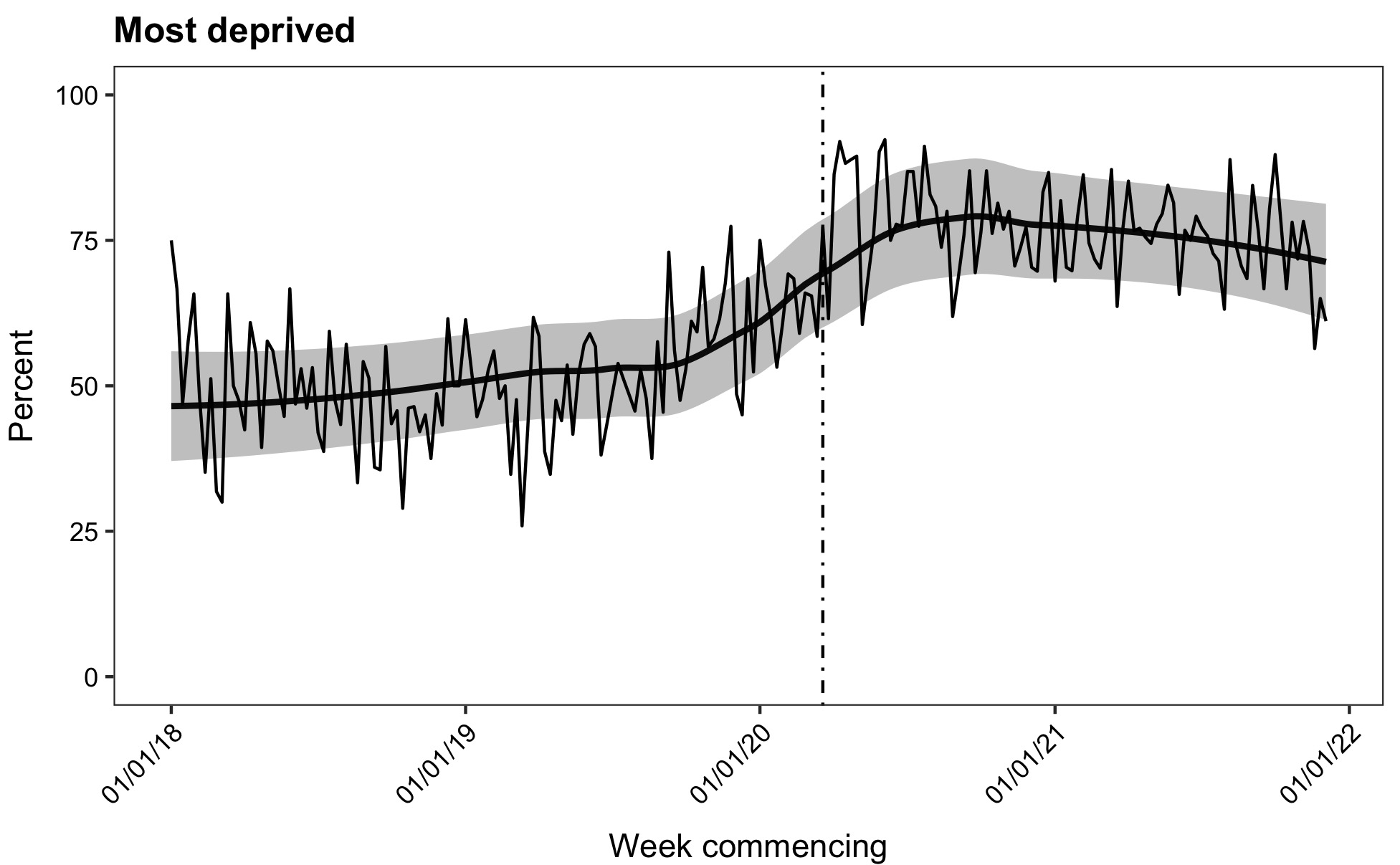   \|  \| IRR \| 95% CI \| SE \| p-value \| \| --- \| --- \| --- \| --- \| --- \| \| Time \| 1.002 \| 1.001-1.003 \| 0.001 \| <0.001 \| \| Level \| 1.382 \| 1.262-1.513 \| 0.047 \| <0.001 \| \| Slope \| 0.997 \| 0.995-0.998 \| 0.001 \| <0.001 \| \| Slope^2^ \|  \|  \|  \|  \| \| Slope^3^ \|  \|  \|  \|  \| |

| **4 weeks quits as a percentage of quit dates set** | |
| --- | --- |
| 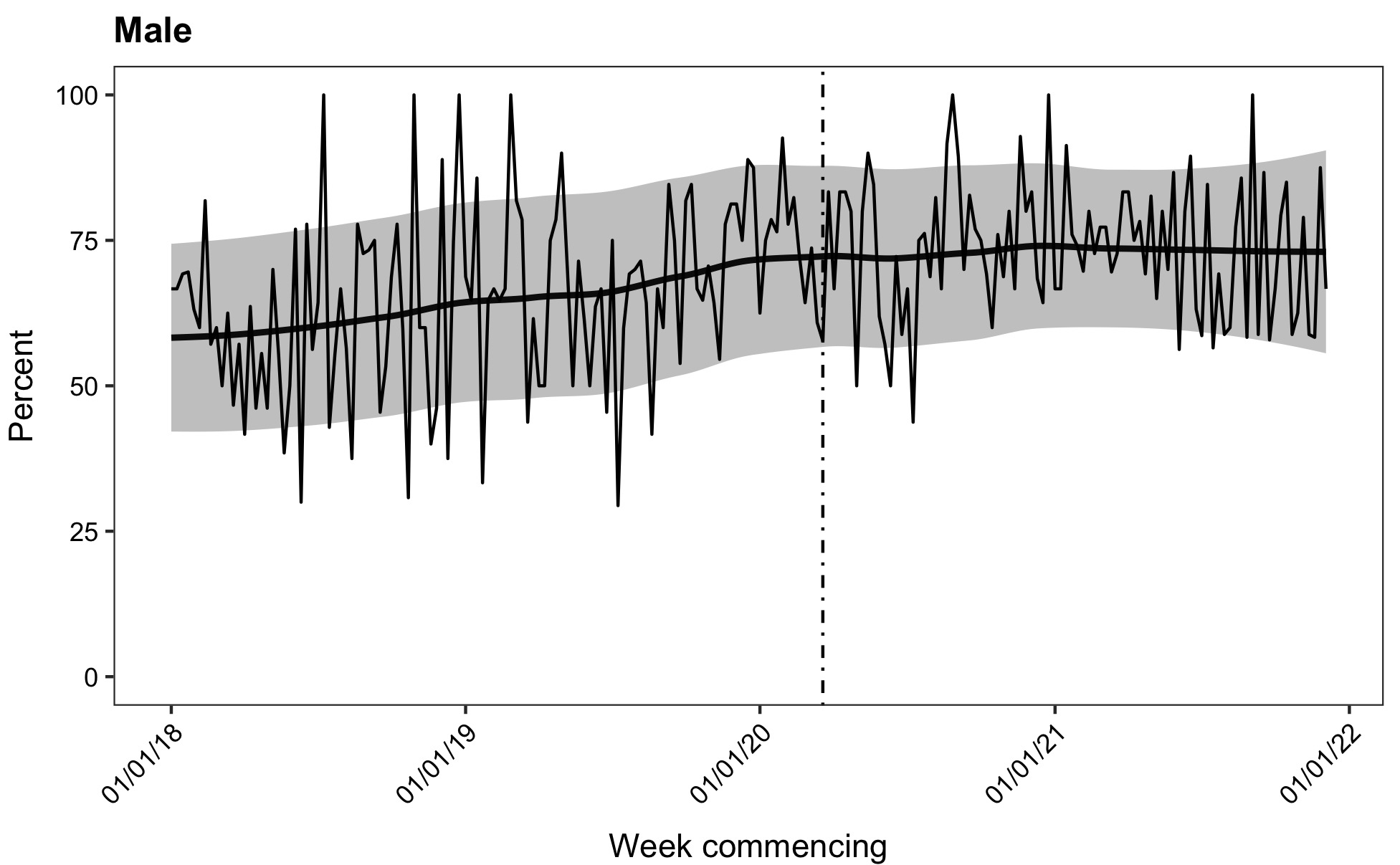   \|  \| IRR \| 95% CI \| SE \| p-value \| \| --- \| --- \| --- \| --- \| --- \| \| Time \| 1.002 \| 1.001-1.003 \| 0.001 \| <0.001 \| \| Level \| 1.005 \| 0.916-1.104 \| 0.048 \| 0.910 \| \| Slope \| 0.998 \| 0.996-1.000 \| 0.001 \| 0.022 \| \| Slope^2^ \|  \|  \|  \|  \| \| Slope^3^ \|  \|  \|  \|  \| | 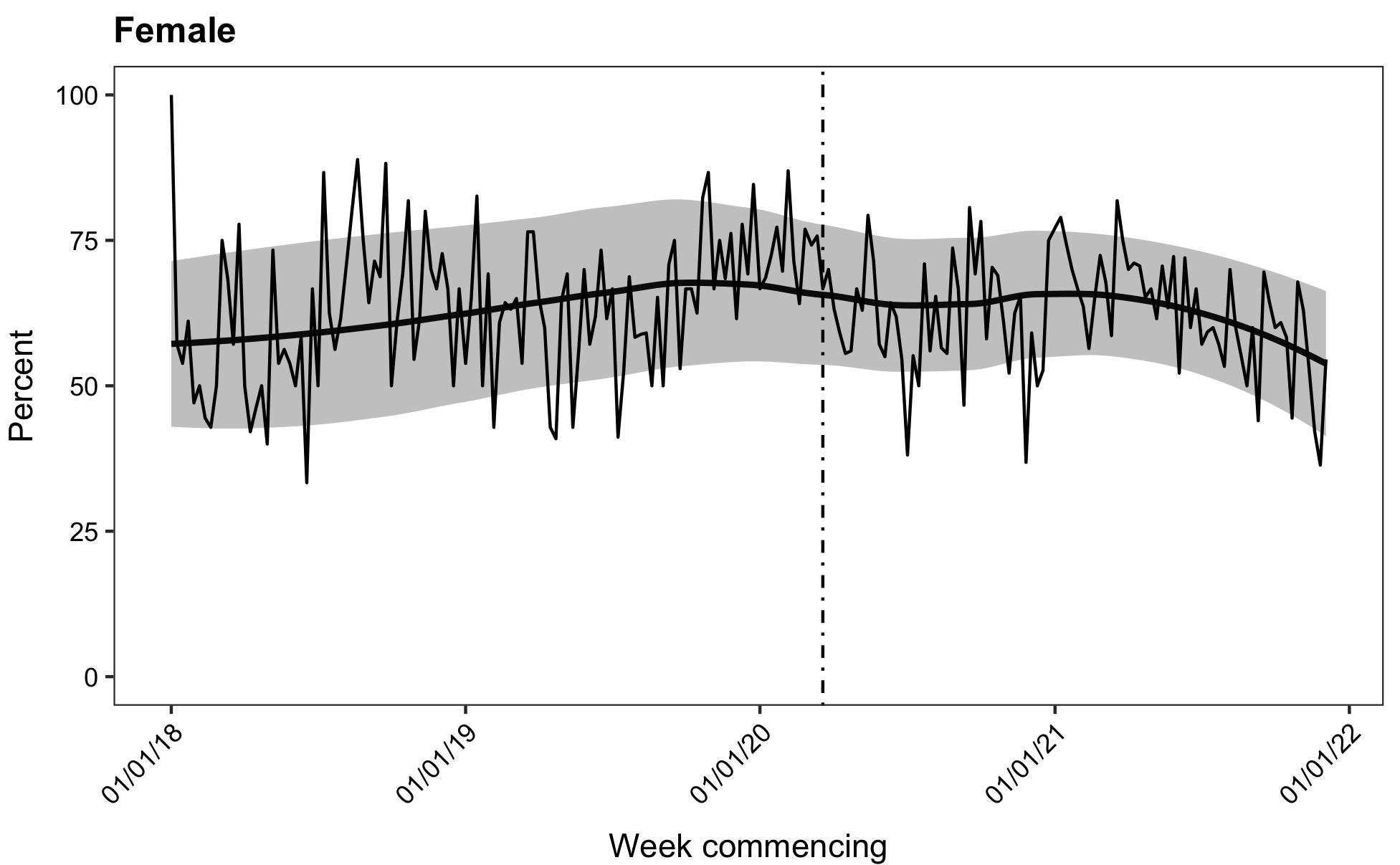   \|  \| IRR \| 95% CI \| SE \| p-value \| \| --- \| --- \| --- \| --- \| --- \| \| Time \| 1.002 \| 1.001-1.003 \| 0.001 \| <0.001 \| \| Level \| 0.820 \| 0.725-0.927 \| 0.063 \| 0.002 \| \| Slope \| 1.004 \| 0.999-1.010 \| 0.003 \| 0.130 \| \| Slope^2^ \| 1.000 \| 1.000-1.000 \| 0.000 \| 0.011 \| \| Slope^3^ \|  \|  \|  \|  \| |
| 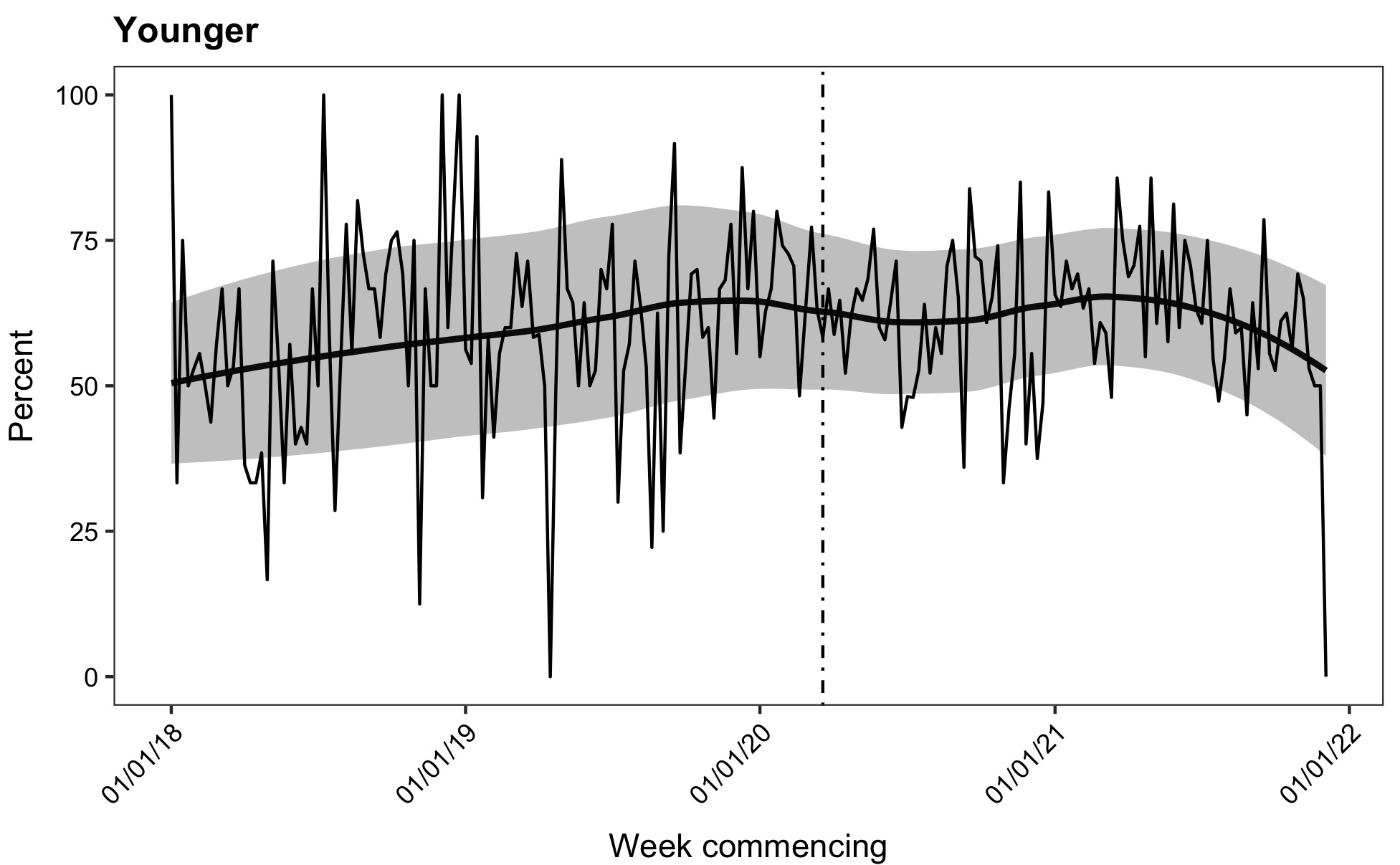   \|  \| IRR \| 95% CI \| SE \| p-value \| \| --- \| --- \| --- \| --- \| --- \| \| Time \| 1.002 \| 1.001-1.004 \| 0.001 \| 0.001 \| \| Level \| 0.961 \| 0.798-1.158 \| 0.096 \| 0.682 \| \| Slope \| 0.987 \| 0.971-1.004 \| 0.009 \| 0.137 \| \| Slope^2^ \| 1.000 \| 1.000-1.001 \| 0.000 \| 0.077 \| \| Slope^3^ \| 1.000 \| 1.000-1.000 \| 0.000 \| 0.036 \| | 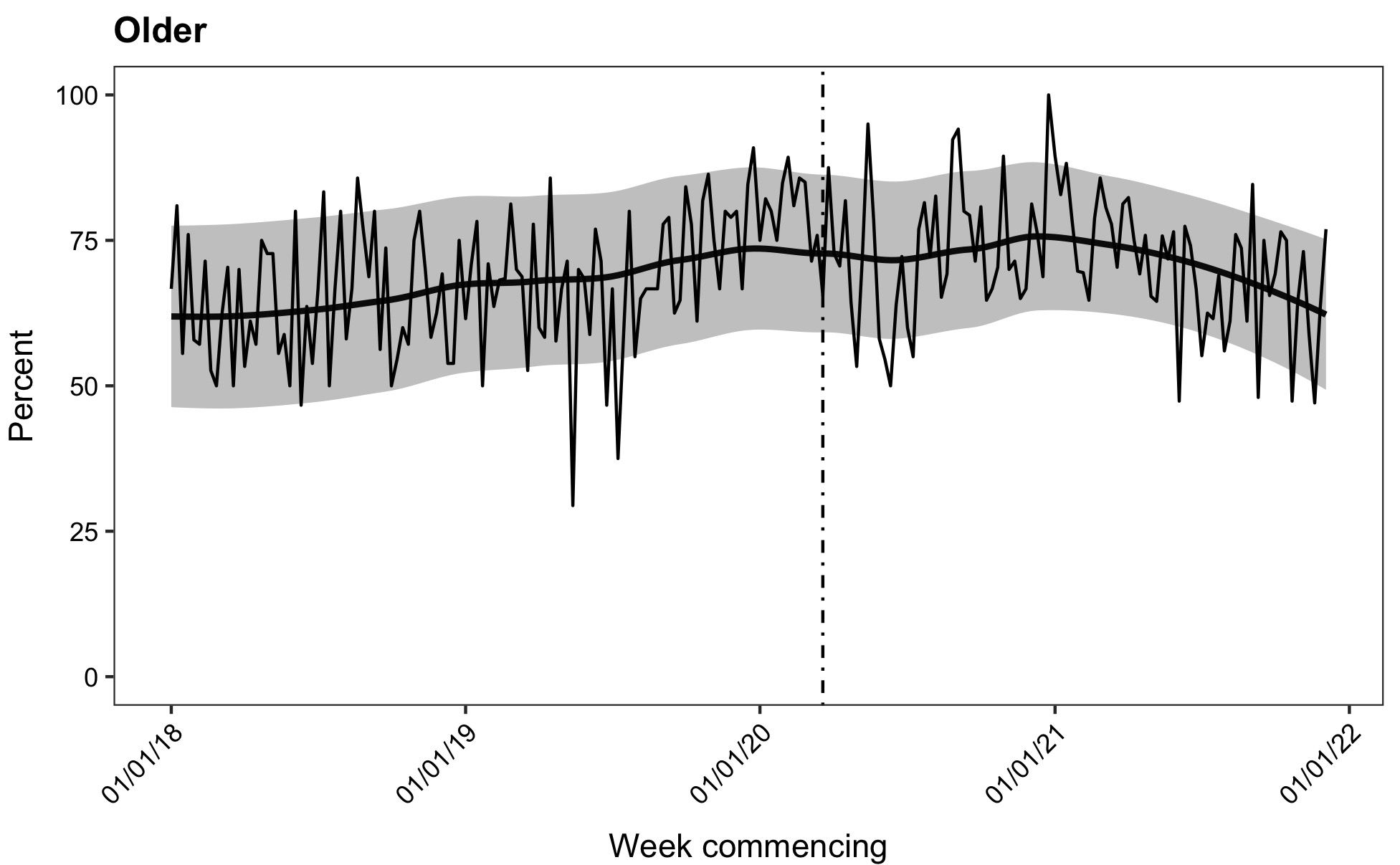   \|  \| IRR \| 95% CI \| SE \| p-value \| \| --- \| --- \| --- \| --- \| --- \| \| Time \| 1.002 \| 1.001-1.003 \| 0.000 \| <0.001 \| \| Level \| 0.916 \| 0.823-1.018 \| 0.055 \| 0.109 \| \| Slope \| 1.003 \| 0.998-1.007 \| 0.002 \| 0.267 \| \| Slope^2^ \| 1.000 \| 1.000-1.000 \| 0.000 \| 0.015 \| \| Slope^3^ \|  \|  \|  \|  \| |
| 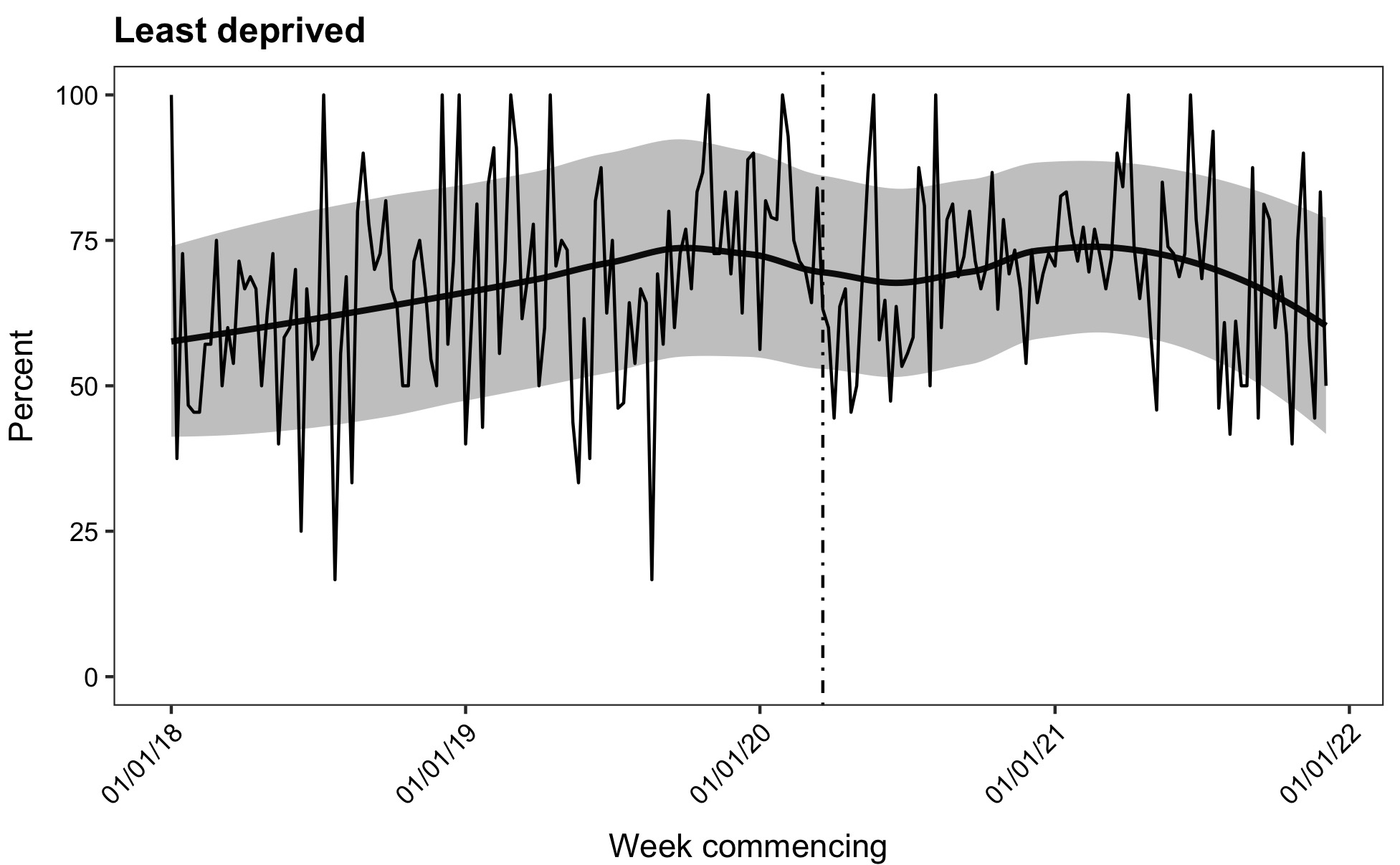   \|  \| IRR \| 95% CI \| SE \| p-value \| \| --- \| --- \| --- \| --- \| --- \| \| Time \| 1.003 \| 1.001-1.004 \| 0.001 \| <0.001 \| \| Level \| 0.744 \| 0.639-0.866 \| 0.078 \| <0.001 \| \| Slope \| 1.008 \| 1.001-1.015 \| 0.003 \| 0.023 \| \| Slope^2^ \| 1.000 \| 1.000-1.000 \| 0.000 \| 0.003 \| \| Slope^3^ \|  \|  \|  \|  \| | 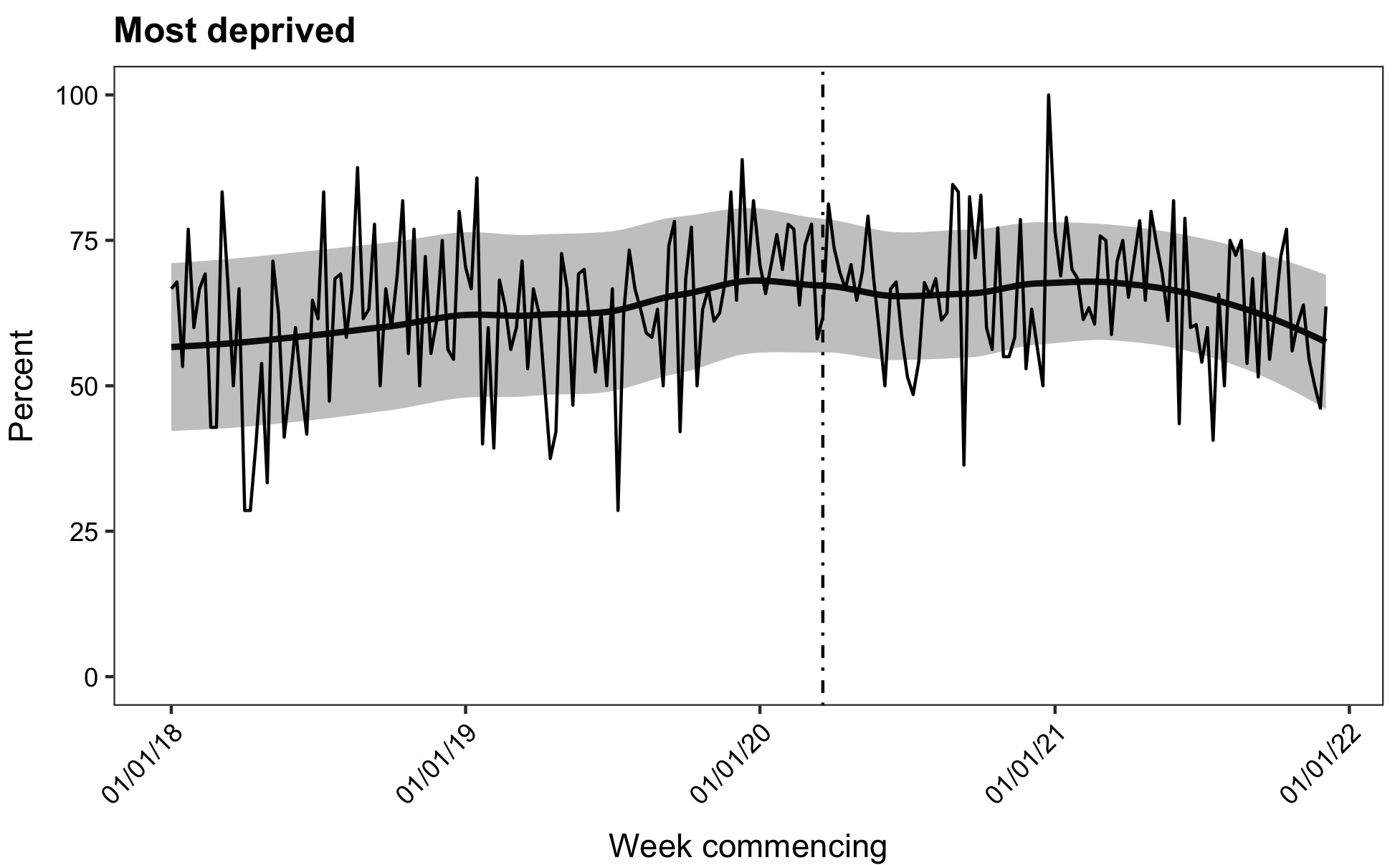   \|  \| IRR \| 95% CI \| SE \| p-value \| \| --- \| --- \| --- \| --- \| --- \| \| Time \| 1.002 \| 1.001-1.003 \| 0.000 \| 0.001 \| \| Level \| 1.089 \| 0.927-1.279 \| 0.083 \| 0.308 \| \| Slope \| 0.985 \| 0.972-1.000 \| 0.007 \| 0.046 \| \| Slope^2^ \| 1.000 \| 1.000-1.001 \| 0.000 \| 0.043 \| \| Slope^3^ \| 1.000 \| 1.000-1.000 \| 0.000 \| 0.026 \| |
